# The Substrate-Driven Transition to an Inward-Facing Conformation in the Functional Mechanism of the Dopamine Transporter

**DOI:** 10.1371/journal.pone.0016350

**Published:** 2011-01-27

**Authors:** Jufang Shan, Jonathan A. Javitch, Lei Shi, Harel Weinstein

**Affiliations:** 1 Department of Physiology and Biophysics, Weill Medical College of Cornell University, New York, New York, United States of America; 2 The HRH Prince Alwaleed Bin Talal Bin Abdulaziz Alsaud Institute for Computational Biomedicine, Weill Medical College of Cornell University, New York, New York, United States of America; 3 Center for Molecular Recognition, Columbia University College of Physicians and Surgeons, New York, New York, United States of America; 4 Department of Psychiatry, Columbia University College of Physicians and Surgeons, New York, New York, United States of America; 5 Division of Molecular Therapeutics, New York State Psychiatric Institute, New York, New York, United States of America; 6 Department of Pharmacology, Columbia University College of Physicians and Surgeons, New York, New York, United States of America; University of South Florida College of Medicine, United States of America

## Abstract

**Background:**

The dopamine transporter (DAT), a member of the neurotransmitter:Na^+^ symporter (NSS) family, terminates dopaminergic neurotransmission and is a major molecular target for psychostimulants such as cocaine and amphetamine, and for the treatment of attention deficit disorder and depression. The crystal structures of the prokaryotic NSS homolog of DAT, the leucine transporter LeuT, have provided critical structural insights about the occluded and outward-facing conformations visited during the substrate transport, but only limited clues regarding mechanism. To understand the transport mechanism in DAT we have used a homology model based on the LeuT structure in a computational protocol validated previously for LeuT, in which steered molecular dynamics (SMD) simulations guide the substrate along a pathway leading from the extracellular end to the intracellular (cytoplasmic) end.

**Methodology/Principal Findings:**

Key findings are (1) a second substrate binding site in the extracellular vestibule, and (2) models of the conformational states identified as occluded, doubly occupied, and inward-facing. The transition between these states involve a spatially ordered sequence of interactions between the two substrate-binding sites, followed by rearrangements in structural elements located between the primary binding site and the cytoplasmic end. These rearrangements are facilitated by identified conserved hinge regions and a reorganization of interaction networks that had been identified as gates.

**Conclusions/Significance:**

Computational simulations supported by information available from experiments in DAT and other NSS transporters have produced a detailed mechanistic proposal for the dynamic changes associated with substrate transport in DAT. This allosteric mechanism is triggered by the binding of substrate in the S2 site in the presence of the substrate in the S1 site. Specific structural elements involved in this mechanism, and their roles in the conformational transitions illuminated here describe, a specific substrate-driven allosteric mechanism that is directly amenable to experiment as shown previously for LeuT.

## Introduction

The dopamine transporter (DAT) is a member of the neurotransmitter:Na^+^ symporter (NSS) family that includes the transporters for other biogenic amines (serotonin and norepinephrine), amino acids (GABA, glycine, proline, taurine) and osmolytes (betaine, creatine) [1]. DAT terminates dopaminergic neurotransmission by transporting dopamine (DA) against its concentration gradient from the synaptic cleft into the pre-synaptic neuron in a Na^+^ and Cl^−^ dependent process. DAT is recognized as the primary target of psychostimulants such as cocaine and amphetamine, and has been implicated in multiple disorders, including Attention-Deficit Hyperactivity Disorder and depression.

Structure-function relations have been studied extensively for DAT using both site-directed and deletion mutagenesis, as well as cross-linking, engineering of metal binding sites, and substituted-cysteine accessibility approaches (see [2]–[9] and references therein). However, the molecular details of the dynamic transport mechanism remain elusive. The high resolution structures of LeuT [10]–[15], a prokaryotic homolog of DAT, have provided essential structural insights that can serve to interpret the results of experimental investigations of DAT in a structural context [16], but offer only limited clues about the molecular mechanism of transport. In the first LeuT structure, the substrate is located in the center of protein, occluded from both the periplasmic and the cytoplasmic milieus. Although the breakthrough structural information about LeuT [10]–[15], and about some other related transporters [17]–[19] is recent, a number of models have been proposed for the functional mechanism of LeuT and cognate NSS transporters. For example, the model proposed by Gouaux et al. involves two additional conformations, outward-facing and inward-facing [15], which is in line with the alternating access model for transporters proposed earlier [20]. Structural modeling supported by experimental probing has offered ideas based on the symmetry features of the molecules in this family [21], [22], and the powerful approach of computational simulation using high resolution structural information was applied to the exploration of the functional mechanisms of proteins with a LeuT-like structure fold (e.g., see [10], [16], [23]–[33]. The current mechanistic understanding emerging from the combined experimental and computational studies, while still incomplete, suggests that the functional mechanisms of the human neurotransmitter transporters in the NSS family are much more complex than would be suspected from the canonical “alternating-access model” of the transition between an outward-facing and an inward-facing form [34].

A central motif of these complex mechanisms is the allosteric effect of ion- and substrate-binding on the translocation process. Both computation and experiment suggest that in LeuT these binding events trigger a series of local perturbations that are propagated from one end of the transporter to the other, generating significant changes in the preferred state [29], [34]. The large-scale structural changes are interpretable as the formation of outward- and inward-open conformations supporting the transport process. One element of the allosteric mechanism that produces the conformational changes through propagation of local perturbations, rather than large rigid body motions, is the effect of ligand binding in the extracellular vestibule of LeuT, termed the S2 binding site [29]. Using binding and flux experiments we had shown that the primary binding site (S1 site) and the S2 site could be occupied by substrate simultaneously, and that substrate in the extracellular vestibule S2 site could allosterically trigger intracellular release of Na^+^ and substrate from the S1 site, thereby functioning as a “symport effector” [29]. The S2 site also binds tricyclic antidepressants (TCAs) [11], which interact differently from the substrate and do not promote substrate release from the S1 site, thereby acting as symport uncouplers that inhibit transport [11]. In addition, we identified from computational analyses of the LeuT structures the nature of rearrangements in the extracellular region that differentiate the actions of substrates from inhibitors bound in the S2 site [10]. The likely structural commonalities among the transmembrane (TM) domains of LeuT and eukaryotic NSS [35], [36] suggest that many of the details elucidated thus far for LeuT will be shared within this protein family. Other details will differ, however, leading to important functional distinctions in selectivity, sensitivity and responses to substrates, ions and various ligands, such as those evidenced among the human neurotransmitter transporters.

For DAT, previous molecular dynamics (MD) simulations have identified structural elements important for substrate binding and the formation of an occluded state [37], [38]. However, the involvement of a LeuT-like S2 binding site and any mechanistic role that an S2-bound substrate might have in modulating DAT function in the manner described for LeuT, remain open questions that are examined here in the context of the allosteric mechanism responsible for conformational transitions in DAT. To study the mechanism of substrate translocation to the intracellular side we used a DAT model described previously [39] to explore the pathway with steered molecular dynamics (SMD) simulations as had been done previously for LeuT [29], [40] and other transporters [33], [41]. Here, this protocol was augmented with the addition of long MD equilibrations of the various DAT states, to determine properties and function-related dynamics of the S1 and S2 sites, as well as the permeation pathway and function-related states of the transporter molecule. We addressed for the first time the (i)-the molecular mechanism of communication between the S1 and S2 sites, and (ii)-the structural and dynamic elements that enable the DAT molecule to open towards the cytoplasm, which allowed us to identify structural elements responsible for the propagation of the conformational changes. These are shown here to consist, respectively, of specific residues positioned between the S1 and S2 sites, and a cluster of aromatic residues positioned below the S1 site toward the intracellular end. The conformational rearrangements are shown to involve specific “hinge residues” in the transition between the occluded and the inward-facing states. We report on remarkable agreement between the identities of the key components in the translocation mechanism we are able to identify from the simulations and experimental data in the literature. Together, these results achieve a comprehensive molecular identification of key elements of the substrate translocation pathway and the underlying allosteric mechanism in DAT, at a level of detail that is directly amenable to further experimental validation.

## Results and Discussion

### I. The S1 and S2 binding sites and the substrate translocation pathway of DAT

**Figure 1:**
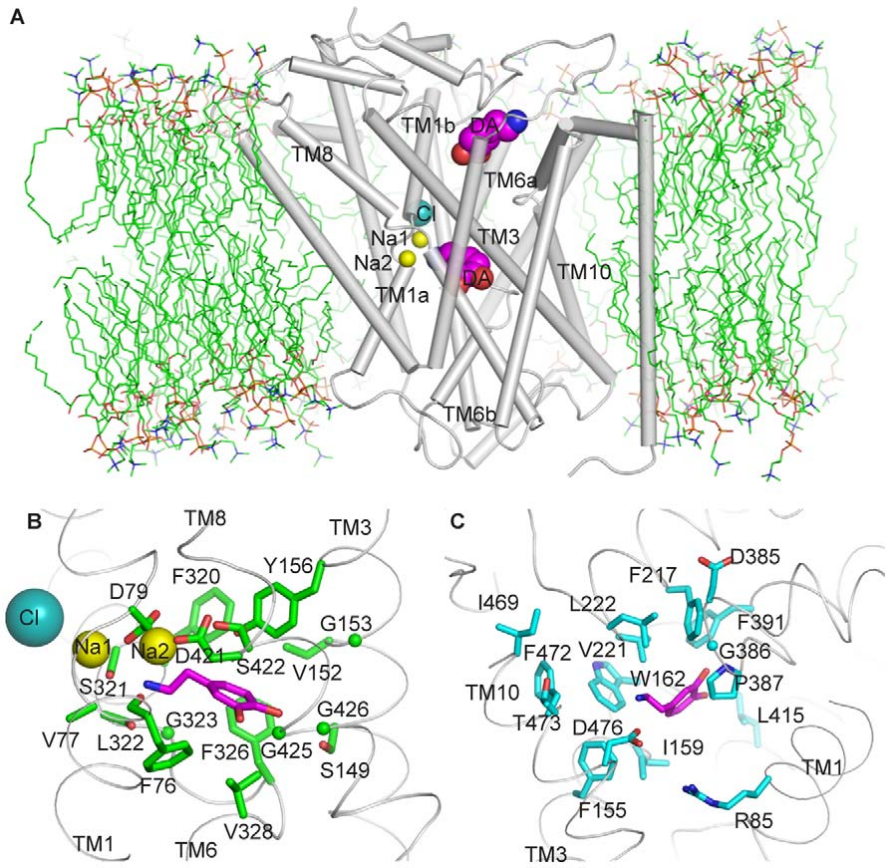
The substrate binding sites of DAT. (**A**) S1,S2-DAT with DA in both the S1 and S2 sites, immersed in a lipid bilayer. The S1 site is located in the middle of the TM bundle and the S2 site is located ∼ 10 Å above the S1 site. (**B**) DA in the S1 site interacts with residues from TMs1, 3, 6 and 8 (viewing perspective is similar to that in (**A**)). (**C**) DA in the S2 site interacts mainly with residues from TMs1, 3 and 10, EL2, and EL4 (viewed from the exit of the extracellular vestibule).

To investigate the translocation mechanism of DAT we performed SMD simulations (see Methods for details) on a homology model that we had constructed previously and simulated in explicit water and lipid environment [39]. This model of DAT in the occluded state (termed here S1-DAT) is based on the LeuT template [15] and the characterization of the Cl^−^ binding site [42], and includes in the S1 site one dopamine (DA) substrate molecule, two Na^+^ and one Cl^−^ ions. DA was docked in the S1 site by aligning its amine and hydrophobic portion with those of the leucine in the crystal structure of LeuT (see Methods for details). The permeation pathway from the extracellular side was explored in this model with SMD simulations pulling DA from the S1 site towards the extracellular side. Much like in LeuT [29], this procedure identified here a second binding site in a region above (extracellular to) the S1 site by the behavior of the steering force experienced when pulling DA that was the same as described previously for the equivalent simulation in LeuT. The force profile (see especially Figure S1 in File S1, in [29]) suggests the presence of an extracellular pocket similar to the S2 site detected computationally and validated experimentally in LeuT at a similar position [29]. After equilibrating a substrate in this S2 site, a second DA molecule was added and positioned in the S1 site, and the dual substrate configuration (S1,S2-DAT) was equilibrated for 25 ns (Figure 1A,C).

To explore the intracellular permeation pathway, two independent SMD simulations were initiated from S1,S2-DAT. After DA from the S1 site reached the intracellular side, the system was further equilibrated with 15 ns MD simulation in each of them. The two separate runs converged to the same final inward-facing conformation. In the following sections, we delineate our characterization of the S1 and S2 sites and the substrate translocation pathway based on the analysis of three equilibrated conformational states, namely, S1-DAT, S1,S2-DAT and the inward-facing conformation.

#### The S1 and S2 binding sites

**Figure 2:**
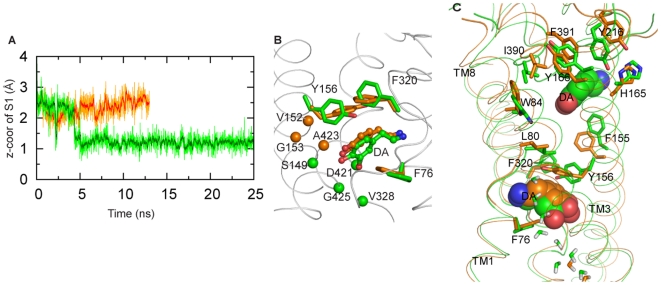
The allosteric effect of S2 on the S1 site. (**A**) Change in position of the DA substrate in the S1 site during equilibration of the S1,S2-DAT model. The green trace shows that the Z-coordinate of DA (center of mass) decreases by ∼ 1.5 Å (i.e., DA is shifted downward toward the intracellular side), compared to its position in S1-DAT (orange trace). (**B**) The positional changes of DA, the rotamer changes of F76^1.42^, Y156^3.50^ and F320^6.53^, and the changes in the composition of the S1 site in S1-DAT (residues rendered in orange) compared to S1,S2-DAT (in green). Note that in S1,S2-DAT, the substrate interacts more (i.e., a higher percentage of time) with S149^3.43^, V328^6.61^, and G425^8.63^, and establishes a new interaction with D421^8.59^ (C_α_ atoms shown as orange spheres) while losing interactions with V152^3.46^, G153^3.47^ and A423^8.61^ (C_α_ atom shown as green spheres). (**C**) The residues forming an interaction network involved in conformational transitions between S1-DAT (orange) and S1,S2-DAT (green). Residues I390^EL4^ and F391^EL4^ are in contact with DA in the S2 site. Note that changes in W84^1.50^, L80^1.46^, Y156^3.50^ and F320^6.53^ are coordinated with I390^EL4^ and F391^EL4^ (Table 1).

The residues forming the S1 site were identified from the equilibration trajectories of both S1-DAT and S1,S2-DAT as those close to the substrate during the simulation trajectories (Figure 1A,B). The resulting binding pose of the DA substrate in the S1 site is consistent with previous studies [37], [38], [43]. Most residues in contact with the S1 substrate were from TMs1, 3, 6 and 8 and remained the same in both trajectories (Figure 1B, Table 1). However, the identities and the orientations of several S1 residues were different when assigned in the presence or absence of substrate in the S2 site (Figure 2B, Table 1), suggesting that S2 binding has an allosteric effect on the S1 site (see below).

**Table 1:** Composition of the S1 site.

Index	Residue	Cα-z^a^	S1-DAT %^b^	S1,S2-DAT %^c^
1.42	F76	−1.24	100	100
1.43	A77	0.78	99	99
1.45	D79	5.32	100	100
3.43	S149	0.39	8	20
3.46	V152	5.11	99	18
3.47	G153	3.19	23	0
3.50	Y156	6.30	95	95
6.53	F320	5.58	100	100
6.54	S321	5.64	100	100
6.55	L322	2.00	99	100
6.56	G323	1.16	100	100
6.59	F326	−1.28	99	98
6.61	V328	−4.64	73	100
8.59	D421	−0.98	0	20
8.60	S422	1.13	100	100
8.61	A423	1.73	24	0
8.63	G425	−3.18	19	100
8.64	G426	−1.17	98	99

a The z-coordinates for residues at the S1 site of DAT. S1-DAT equilibrated at 14 ns was used for the analysis since it is the starting structure for pulling DA towards the extracellular side.

b The percentage of time spent by the residue within 3.5 Å of DA (see Methods) during the 6–16 ns segment of equilibration of the S1-DAT model.

c The percentage of time spent by the residue within 3.5 Å of DA during the 6–25 ns segment of equilibration of the S1,S2-DAT model.

The S2 site residues identified in the equilibration trajectory of S1,S2-DAT were from TMs1, 3, and 10, and the extracellular loops EL2 and EL4 (Figure 1C, Table 2). The composition of the S2 site in DAT is similar to that in LeuT [29], and includes the corresponding (aligned) hydrophobic residues F155^3.49^, I159^3.53^, W162^3.56^, and F472^10.44^ and a pair of corresponding charged residues, D476^10.48^ and R85^1.51^. Note, however, that EL2 is much longer in eukaryotic NSS than in LeuT, and is involved in the S2 site of the DAT model, but not in the S2 site of LeuT.

**Table 2:** Composition of the S2 site.

Index	Residue	Cα-z^a^	S1,S2-DAT-Average %^b^	Inward-Average %^c^
1.51	R85	14.00	30	1
3.49	F155	8.26	90	92
3.53	I159	10.20	45	97
3.56	W162	13.55	93	99
EL2	F217	25.71	51	94
	V221	21.92	98	98
	L222	23.71	94	53
EL4	D385	26.11	22	0
	G386	22.68	31	2
	P387	20.82	80	100
	F391	23.47	19	4
8.53	L415	11.26	3	98
10.41	I469	20.78	30	34
10.44	F472	16.09	71	0
10.45	T473	18.04	49	0
10.48	D476	14.19	99	100

a as in Table1.

b The percentage of time spent by the residue within 3.5 Å of DA (see Methods) during the 6–25 ns of equilibration of the S1,S2-DAT model.

c The percentage of time spent by the residue within 3.5 Å of DA during the 6–15 ns of equilibration of the inward-facing model.

#### The permeation pathway

Residues contacted as the substrate moved from the S1 site outward toward the S2 site during the SMD/MD simulation were classified as belonging to the extracellular transport pathway that is lined by two layers of hydrophobic residues along TMs1, 3, 8 and 10 (Table 3). Following the same criterion, residues in contact with the substrate as it moved from the S1 site toward the cytoplasmic side in the two independent SMD simulations, were similarly classified as belonging to the intracellular translocation pathway lined mainly by residues from TMs1, 5, 6 and 8 (Table 4).

**Table 3:** The substrate translocation pathway from the S1 site to the S2 site.

Index	Residue	Cα-z^a^	Max %^b^
1.46	L80	8.96	100
1.47	A81	9.50	100
1.50	W84	14.00	55
3.57	A163	12.42	27
EL4	I390	20.48	10
10.49	H477	14.84	5
10.51	A479	10.00	76
10.52	A480	12.12	100

a as in Table 1.

b For SMD calculations, percentages were defined as the number of frames during which substrate sees a residue/(Frame No. when it last sees that residue - Frame No. when it first sees that residue); both the SMD and MD equilibrations were included for the calculation. The maximum percentages from individual SMD or MD simulations are reported.

**Table 4:** The substrate translocation pathway from the S1 site to the intracellular site.

Index	Residue	Cα-z a	Max (1^st^/2^nd^) % b
1.25	R60	−20.47	69/44
1.29	W63	−14.34	99/94
1.38	S72	−6.59	1/100
1.41	G75	−1.70	1/24
2.66	A128	−9.93	56/25
4.62	L255	−7.14	59/0
4.65	G258	−10.61	0/35
5.36	V259	−11.44	98/70
5.40	S262	−7.22	63/81
5.41	G263	−8.90	16/27
5.43	V266	−3.55	94/97
6.62	L329	−6.63	16/0
6.64	A331	−9.52	100/100
6.65	F332	−9.81	100/100
6.68	Y335	−15.58	100/100
8.62	M424	−1.90	51/47
8.66	E428	−6.11	100/100
8.67	S429	−5.48	78/84
8.70	T432	−8.97	3/66
8.71	G433	−9.56	11/58
8.74	D436	−14.31	7/44
9.38	R445	−13.45	1/50

a as in Table 1.

b as in Table 3. The results are shown as the percentage from the first simulation/percentage from the second simulation.

The endpoint for SMD pulling in the intracellular pathway was determined by monitoring the interaction energy between DA and solvating water molecules. In the S1 site the water-DA interaction energy was ∼ −19 kcal/mol, reflecting minimal direct contact. The water-DA interaction became stronger as the substrate moved toward the cytoplasm, indicating increasing solvation until DA established an equilibrated interaction with the conserved E428^8.66^, when the interaction energy stabilized at ∼ −60 kcal/mol suggesting full solvation by surrounding waters; this was supported by visual inspection. SMD pulling was terminated at this position. Notably, the residue corresponding to E428^8.66^ in various transporters has been shown to be important for substrate transport [44], [45] and shown to become solvent exposed in the inward-facing conformation of GAT-1 [45]. The observed interaction between DA and E428^8.66^ suggests that this functionally important glutamate may be an anchoring point along the transport pathway where the substrate makes stable interactions before it moves to the cytoplasm, or reversely in the initial step of efflux.

### II. Substrate binding in the S2 site prepares DAT for the transport of dopamine

The changes observed in the S1 site when substrate is present in the S2 site suggest a mode of allosteric interaction between the binding sites. The elements of this allosteric mechanism are the coordinated rearrangements of key residues and structural elements (TM segments and loops) discussed below.

#### An allosteric interaction network between the S1 and S2 sites

The presence of substrate in the S2 site is associated with a downward repositioning of the center of mass of the S1-bound substrate by 1 Å (Figure 2A,B). This is accompanied by the dissociation of several DA-protein contacts, and the formation of new ones (Figure 2B). Comparison of the equilibrated S1-DAT and S1,S2-DAT models showed that the downward repositioning is enabled by the rearrangement of (i)-several conserved hydrophobic residues within the S1 site, or in between the S1 and S2 sites (e.g., the significant changes of F76^1.42^, Y156^3.50^ and F320^6.53^ shown in Figure 2B), and (ii)-the interposed water molecules. The rearrangement involved in the S1/S2 allosteric interaction is accomplished by a set of highly conserved residues that are not sequential in the primary structure, but form a network in space as indicated in Figure 2.

Compared to S1-DAT, which corresponds to the LeuT crystal structure, in the S1,S2-DAT model the EL2 and EL4 loops have moved towards the S2 substrate and pushed the extracellular segment of TM3 inward to the S2 site (see Figure S1 in File S1). This appears to cause conformational changes in the S1 site, propagated through a conserved interaction network (Table S1 in File S1). The dynamic sequence of events has I390^EL4^ and F391^EL4^ pushing the sidechain of W84^1.50^, and then of L80^1.46^ down toward the S1 site (Figure 2C). As a result, the sidechains of Y156^3.50^ and F320^6.53^ that are located between the S1 and S2 sites and have their phenyl rings above the catechol moiety of DA, rotated to push downward on the ligand in the S1 site. F155^3.49^ facilitated the rotation of Y156^3.50^ in a rearrangement that is likely due to its interaction with the substrate in the S2 site. Thus, the communication between the S1 and S2 sites appears to depend on this sequence of rearrangements of the highly conserved set of hydrophobic and aromatic residues (L80^1.46^, W84^1.50^, F155^3.49^, Y156^3.50^, F320^6.53^, I390^EL4^ and F391^EL4^). Indeed, the difference distance matrix map (Fig. S1 in File S1) shows that residues L80^1.46^, W84^1.50^, I390^EL4^ and F391^EL4^ moved together with F155^3.49^, Y156^3.50^ or F320^6.53^. This type of allosteric communication function carried out by a conserved network in the molecular space had been proposed for allosteric networks in other molecular systems as well, e.g., in PDZ domains and other proteins [46]–[48].

The functional role of the rearrangements we observed extends to a change in the coordination of Na2 in the S1,S2-DAT model, which may well be associated with the release of the ion in a process observed both computationally and experimentally for LeuT, as described in [29]. Compared to the S1-DAT, in the S1,S2-DAT model the backbone carbonyl of L418^8.56^ is flipped, with a 130° change in its ψ angle (Table S1 in File S1), so that it no longer coordinates Na2 (Figure S1G in File S1) but interacts instead with W84^1.50^ (through a water molecule), which stabilizes it in a new position. With this set of rearrangements, the middle portion of TM8 near L418^8.56^ moves back from the Na2 site and away from TM1, facilitating the observed rotamer changes in L80^1.46^ and Y156^3.50^.

#### The allosteric effect of S2 binding induces an open-inward conformation allowing water penetration

**Figure 3:**
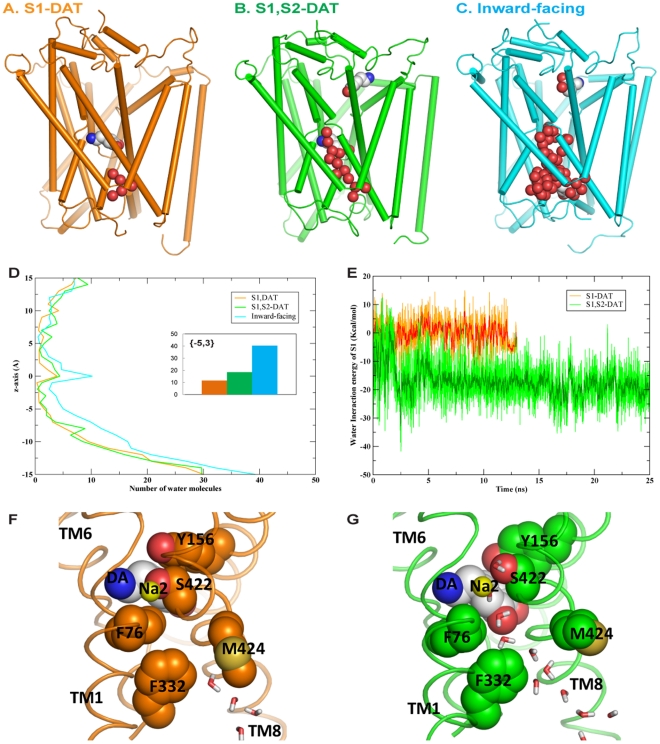
The S2-induced transition of DAT to an inward-open conformation is accompanied by water penetration. (**A**, **B**, **C**) Waters (red balls) gradually penetrate to the S1 site, during the transition from S1-DAT (**A**) to S1,S2-DAT (**B**), then to the inward-facing conformation (**C**). (**D**) Average number of waters along the membrane normal (the z-axis; z = 0 is at the center of the membrane) in (**A**)–(**C**). The insert shows waters accumulated between −5 Å and +3 Å (z-axis) in the corresponding models identified by the colors. (**E**) DA in the S1 site interacts more favorably with waters in the presence of a substrate in the S2 site (green) than in the absence of a substrate in the S2 site (orange). (**F**) and (**G**) show magnified details of (**A**) and (**B**), respectively, with the same color coding and water in stick representations.

We found that the formation of continuous water pathways in the core of DAT (Figure 3) is associated dynamically with substrate binding in the S2 site. The pathway determined from the SMD simulations for the exit of substrate from the S1 site corresponds exactly to a water channel (Table 4), thus identifying a specific mechanism for substrate binding in the S2 site to trigger permeation. Notably, the residues in the highly conserved aromatic cluster lining this channel, which we observe in the simulations to coordinate the movement of DA, are buried in the core of the transporter in the S1-DAT state. However, when S2-bound DA triggers the formation of the channel, part of this aromatic cluster (F69^1.35^, F76^1.42^ and F332^6.65^) becomes more solvated (Figure S2 in File S1), supporting the relation between substrate binding in the S2 site and the opening of the intracellular pathway for the penetration of water and the downward movement of the substrate from the S1 site. Correspondingly, the interaction energy of the S1-bound substrate with water is ∼ −10 kcal/mol in the absence of S2-bound substrate, but becomes much stronger (∼−19 kcal/mol) when substrate occupies the S2 site due to water penetration that also facilitates the process of release into the cytoplasmic medium (Figure 3E). This is enabled by rotamer changes of the buried S262^5.40^ and M424^8.62^ residues in the core of the protein in S1,S2-DAT (Table S1 in File S1), which make room for waters to move up from the intracellular side (Figure 3F,G). Further, the change in conformation of F76^1.42^ (see above) induced S422^8.60^ to move toward Y156^3.50^ (Figure 3F,G), resulting in the disruption of the interaction between S422^8.60^ and F76^1.42^ and water penetration into the S1 site. Notably, Na2 is solvated in this process (facilitating its inward release as described in our findings for LeuT [29]), whereas Na1 remains fully secluded from water.

#### Release of DA from the S1 site involves specific changes in a conserved cluster of aromatic residues

**Figure 4:**
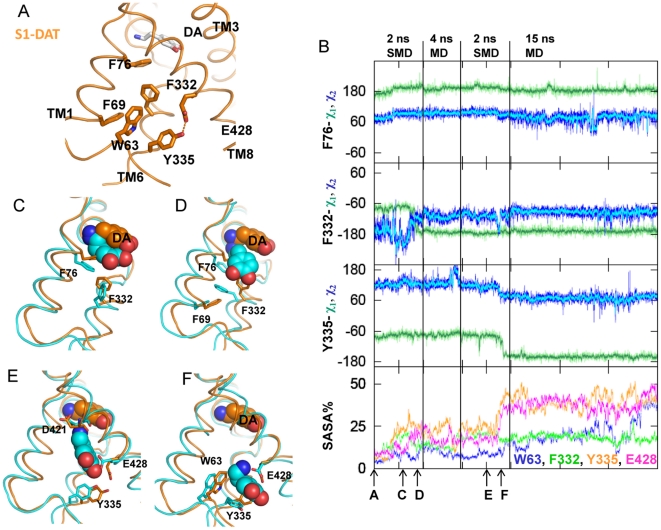
Conformational changes in the aromatic cluster during DA movement inward from the S1 site. (**A**) The cluster of aromatic residues from TMs 1 and 6 shown in S1-DAT is important for conformational transitions; DA in the S1 site is rendered in stick representation. Orange dashed line indicates H-bond of Y335^6.68^ to E428^8.66^. (**B**) Time evolution of dihedrals and SASA (bottom panel) during inward pulling in SMD and MD alternation. Time points marked by the A, B, C, D, E, F arrows on the x-axis correspond to the structures shown in the (**A**)**–**(**F**) panels. The lines in the SASA plot are coded in colors corresponding to the residues names in the same colors. (**C**) The change in the rotamer of F76^1.42^ from the conformation in S1-DAT (orange) to the configuration at the time point indicated by the C arrow in (**B**) (cyan), which allows the downward movement of DA. (**D**) The subsequent change in the rotamer of F332^6.65^ (same color coding as in (**C**)) as DA moves to the position originally occupied by the sidechain of F332^6.65^. (**E**) When DA is slowly pulled down a bit further in the SMD protocol, its amine forms a new H-bond with the carboxyl oxygen of D421^8.59^ which coordinates Na2 in S1-DAT, and its hydroxyl groups forms H-bonds with the sidechain carboxyl group of E428^8.66^. Time is indicated by the E arrow in (**B**). (**F**) The rotamer of Y335^6.68^ changes last, breaking the H-bond between Y335^6.68^ and E428^8.66^. DA moves to the position originally occupied by the sidechain of Y335^6.68^ and E428^8.66^. Time is indicated by the F arrow in (**B**).

We found that ligand binding in the S2 site triggers remarkable changes in the putative permeation pathway of DAT, but the subsequent inward movement of DA from the S1 site toward the intracellular exit involves additional conformational rearrangements in a cluster of highly conserved aromatic residues in TMs1a and 6b (Table S2 in File S1) that includes F69^1.35^, F76^1.42^, F332^6.65^, as well as W63^1.29(NT)^ and Y335^6.68^ (Figure 4A). A set of sequential rotamer changes in these residues is propagated through a series of local conformational rearrangements in the intracellular segments of the TMs. The changes in the rotamers of F76^1.42^, F332^6.65^ and Y335^6.68^ along the translocation pathway (Figure 4B), are correlated (Figure S3 in File S1), as indicated by the application of Spearman's rank test [49] to the trajectory-derived data from the SMD simulations of substrate moving inward from the S1 site. Specifically, rotamer changes in F76^1.42^ and F332^6.65^ allow DA to exit from the S1 site (Figure 4C,D), and rotation of F332^6.65^ produces the rearrangement of F69^1.35^ that results in the significant movement of the intracellular end of TM1a away from TM6b (Figure 4D). Finally, Y335^6.68^ dissociates from an H-bond interaction with E428^8.66^, enabling further movement in TM1a and the N-terminus, which includes W63^1.29(NT)^ (Figure 4E,F). Overall, the conformational changes within the entire aromatic cluster produce an opening surrounded by TMs 1, 5, 6 and 8 that enables water penetration, as indicated by the significant increase in the values of solvent accessible surface areas (SASA) calculated for the dynamics trajectories for residues F332^6.65^, Y335^6.68^ and W63^1.29(NT)^ in a time sequence corresponding to the direction of the pathway (Figure 4B).

The dynamic mechanism emerging from our SMD and MD simulations indicated that the rearrangements of F76^1.42^, F332^6.65^ and Y335^6.68^ in the aromatic cluster, together with E428^8.66^, function as “gates” along the intracellular translocation pathway. The first of these gates is composed of F76^1.42^ and F332^6.65^ (Figure 4C,D) and thus hydrophobic in nature. When the sidechains of the two residues rotate away from the translocation pathway, DA is able to move downward and leave the S1 site. The second gate involves Y335^6.68^ and E428^8.66^ connected by an H-bond. When Y335^6.68^ rotates and the H-bond breaks (Figure 4E,F), DA can move further down the intracellular end of the DAT protein, where it becomes fully solvated. These multi-residue gates open due to coordinated changes in the rotamers of the residues in the aromatic cluster (discussed above) and their neighbors, underscoring the key role of this highly conserved cluster of aromatic residues (W63^1.29(NT)^, F69^1.35^, F76^1.42^, F332^6.65^ and Y335^6.68^) in the conformational transition between different functionally-related states of the transporter protein. This is similar to the aromatic cluster found in TMs 5–6 of Class A GPCRs that is known to contribute to switching between inactive and active states of the receptor (e.g., see [50]–[52]).

### III. Global rearrangement from S1-DAT to the inward-facing conformation

The ordered sequence of local structure perturbations described above gives rise to a global conformational rearrangement of the transporter molecule from one state (e.g., S1-DAT) to another (e.g., inward-facing). A structural characterization of this transition to the inward-facing conformation was obtained with the RMSDTT algorithm [53] that performs an iterative alignment of structures, giving larger weight to regions that have small residue-based RMSDs. Comparing S1-DAT to the inward-facing conformation, this procedure showed that as the intracellular passage opened for the substrate DA, portions of TM segments positioned extracellular to the S1 site have small residue-based backbone RMSDs (1–2 Å), whereas the TM segment portions ranging from the S1 site downward to the intracellular side exhibited drastic conformational changes, with RMSDs of 3–6 Å (Figure S4 and Table S3 in File S1). The same structural characterization of the transition was observed when the models of the two DAT states were aligned using two other approaches, either aligning residues in the conserved TMs1, 3, 6 and 8, or performing 3-D structural alignment of entire DAT structures (“Stamp structural alignment” in VMD [54], [55]).

**Figure 5:**
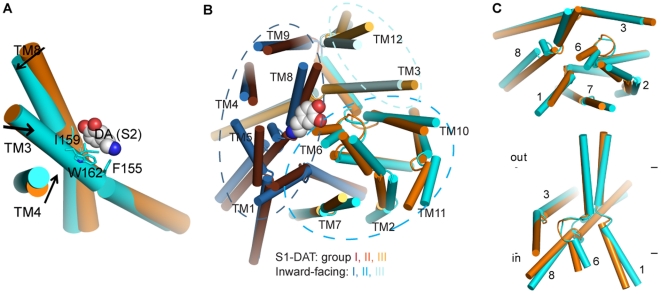
The global rearrangement from S1-DAT to the inward-facing conformation. (**A**) The global rearrangement of TMs3, 4 and 8 at the extracellular side, from S1-DAT (orange) to the inward-facing conformation (cyan). Proteins are aligned with RMSDTT [53] using the entire models. In the inward-facing conformation, TM3 residues F155^3.49^, I159^3.53^ and W162^3.56^ (in sticks) are in direct contact with DA (in spheres, colored by atom type) in the S2 site. (**B**) Viewed from the intracellular side, the movement of the 12 intracellular TM segments (rendered in cartoon) can be partitioned into three groups as indicated by the dotted circles (see text). During the transition to the inward-facing conformation, the first group (colored in chocolate brown for S1-DAT and blue for the inward-facing conformation) moves outward; the second group (orange for S1-DAT and cyan for the inward-facing conformation) moves outward and away from the first group; the third group (yellow for S1-DAT and pale cyan for the inward-facing conformation) moves inward. DA is rendered in spheres and colored by atom types. (**C**) The global movement of selected TMs viewed from the intracellular side (top panel) and parallel to the membrane (bottom panel). During the transition, the extracellular ends of segments TM1b and TM6a do not move, while the intracellular end segments TMs1a and 6b swing away, non-symmetrically, from TMs 3 and 8 to open the substrate translocation pathway. TM1a moves substantially more, thereby distancing itself from TM6b.

The global conformational rearrangement at the extracellular side caused by the S2-bound substrate is small, but noticeable. TM3 residues F155^3.49^, I159^3.53^ and W162^3.56^ are in direct contact with the ligand in the inward-facing conformation and alter the nearby TM packing, so that TM4 moves toward TM3, and TM8 tilts outward to TMs3 and 4 (Figure 5A). Both EL2 (connecting TMs3 and 4) and EL4 (connecting TMs7 and 8) move downward to interact with the ligand and close the S2 site. In addition, EL3 (between TMs5 and 6) also moves inward toward the bundle, consistent with the movement of EL3 observed in LeuT [10], [34], [56].

Conformational rearrangements at the intracellular side make room for the descending DA: TMs1, 4, 5, 8, and 9 move as one group, and TMs2, 6, 7, 10, and 11 as a second group that distances itself from the first. Notably, TM1 exhibits the largest movement, consistent with experimental data for LeuT [34]. TMs3 and 12 move to fill in the space created by the rearrangements of the two groups (Figure 5B), so that TM3 moves towards the position originally occupied by the second group to maintain its associations with TMs6 and 10, and TM12 to maintain contacts with TMs3, 8 and 9.

Overall, we found the global rearrangements in DAT to be similar to those observed from the comparison of the LeuT crystal structure of the occluded form [15] to the inward-facing LeuT model obtained from SMD [57], with the difference that in LeuT some intracellular portions of TM segments exhibited either smaller-scale movements (TMs4 and 8) or did not move at all (TMs3, 9 and 10). This difference may well be due to the higher rigidity that LeuT, which is from a hyperthermophilic organism, would be expected to exhibit at the simulated room temperature; the rigidity is likely to be achieved by a combination of several factors [58].

#### Hinge regions enable the global conformational transitions

That specific hinge regions enable the dynamics of propagation of the observed allosteric effects was established first for the unwound regions of TMs1 and 6. In the transition from S1-DAT to the inward-facing conformation, the extracellular end segments of TM1 (TM1b) and TM6 (TM6a) (above the unwound region) remain largely unchanged, whereas the intracellular ends, TMs1a and 6b, swing outward non-symmetrically to open the substrate translocation pathway. TM1a moves substantially more, thereby distancing itself from TM6b (Figure 5C). Consistent with the mechanism proposed from the crystal structure of LeuT [15], we did not observe a big movement in TMs1b and 6a in the transition between S1-DAT and the inward-facing conformation. Interestingly, comparing LeuT structures in an occluded state [15] and in an outward-facing state [12], it is the intracellular segments TMs1a and 6b that were considered to remain immobile, while the extracellular TMs1b and 6a tilt outwards. The findings described here are also consistent with previously observed changes in the Tyt1 transporter upon opening of the translocation pathway, where this opening is associated with increased solvent accessibility of C18^1.39^ and C238^6.65^ [28]. The increased solvent accessibility at these two positions in Tyt1 was attributed to the rearrangement in TMs1a and 6b in the inward-facing conformation [28]. In the inward-facing conformation of DAT, TMs1a and 6b also move away from each other and the corresponding residues at the two positions, V73^1.39^ (data not shown) and F332^6.65^ (Dehnes et al, manuscript in prep), become more solvent accessible. These results, together with the corresponding observations in LeuT, support the view that TMs1 and 6 do not rock like a rigid bundle [21] when the transporter converts from the outward-facing to occluded conformation, and then to the inward-facing conformation (see Concluding Remarks). Indeed, the rearrangements of TMs1b-6a and TMs1a-6b appear to be driven by different local rearrangements and are thus separated during the transport cycle.

Other types of hinge regions comprise one or more conserved Gly/Pro/Thr/Ser/Cys residues (e.g., G55^2.48^, P57^2.50^, G294^7.45^, G408^10.52^ and P457^11.50^) that enable rigid-body motions of helical segments. Using the Prokink analysis tool [59] implemented in the Simulaid suite [60] we found that for most TMs, the bend angle and face shift values produced by TM helix breakers change significantly in the transitions (Table 5). These local conformational changes connect individual TMs to the configuration changes propagated from the S2 to the S1 site, and further from the S1 site to the intracellular side of the transporter (see Text S2 and Figures S5, S6, S7 within File S2).

**Table 5:** ProKink analysis.

Residues	bend angle				face shift				References
	S1-DAT		inward-facing		S1-DAT		inward-facing		
	Avg.	std	Avg.	std	Avg.	std	Avg.	std	
L80^1.46^	32.8	2.3	38.5	2.7	127.3	6.6	165.0	5.8	[77], [78]
P101^2.39^	12.0	3.9	19.4	3.1	26.8	13.1	9.9	11.1	[5]–[9]
P112^2.50^	19.3	3.0	24.1	2.7	117.7	40.2	9.1	144.5	[5], [6], [9], [79]
S149^3.43^	22.0	2.8	14.5	2.7	13.3	6.1	13.7	6.3	[80]
G153^3.47^	10.2	3.7	19.4	3.7	18.4	7.4	14.7	6.8	[80]
S254^4.61^	43.2	2.8	55.0	3.4	10.0	10.5	41.8	8.9	[81]–[83]
T269^5.46^	6.0	3.3	15.8	4.0	−13.5	5.4	15.7	7.0	[82], [84]
P273^5.50^	22.3	5.2	24.2	5.1	59.5	20.3	58.2	18.3	[5], [6], [84], [85]
S354^7.39^	6.2	2.2	6.0	2.6	8.0	6.8	12.1	5.5	[82], [86]–[89]
S357^7.42^	6.2	2.0	6.0	1.9	21.5	7.7	12.7	6.3	[82], [88], [90]–[92]
S422^8.60^	14.9	3.1	14.2	3.1	0.1	8.6	44.3	9.4	[83], [93]
G426^8.64^	17.1	5.2	11.9	4.8	18.9	14.8	25.3	13.2	n/a
T456^9.49^	6.2	2.7	7.1	2.8	32.1	7.4	26.8	8.0	[82], [87]
S460^9.53^	5.9	3.2	6.6	3.4	15.8	8.4	17.7	8.3	[82], [94]
G481^10.53^	31.6	2.4	36.6	2.2	59.2	8.4	57.6	16.3	n/a
S483^10.56^	30.4	3.1	33.7	2.1	137.1	4.5	80.5	4.0	[82], [95], [96]

Experimental support for these observations was obtained from the rich structure-function information in the literature collected through the TRAC information management platform [57]. The results show that mutations of corresponding residues in these hinge regions in various NSS transporters disrupt the binding profile and/or the translocation cycle, as indicated by decreased binding affinities of substrates and ligands, increased K_m_ and/or decreased rates of transport, or altered DA efflux (see Table 5 and references therein). This underscores the functional importance of conformational changes associated with “hinges” in the course of the transition between functional states of the transporter.

#### Interaction networks are reconfigured in the transition

**Figure 6:**
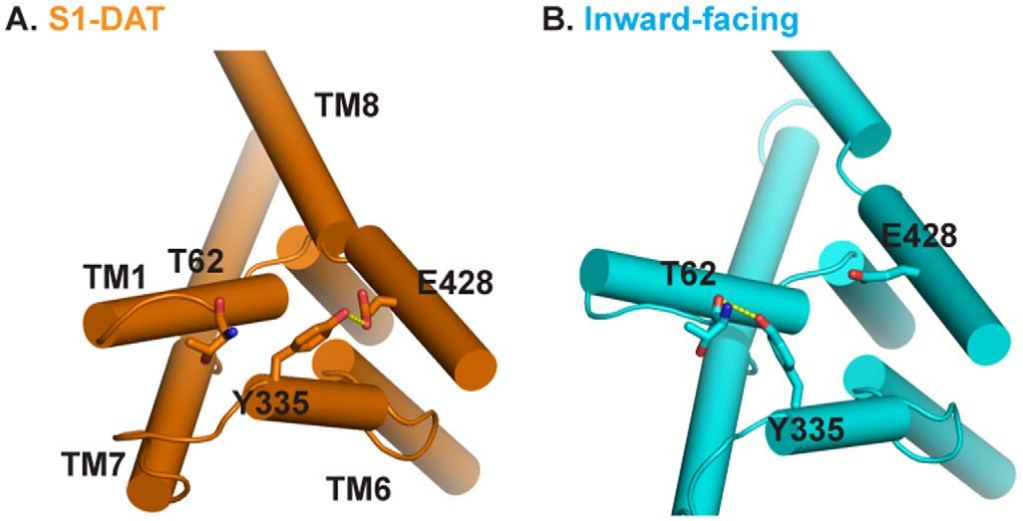
Changes in intracellular interaction networks. Y335^6.68^ forms an H-bond with E428^8.66^ in S1-DAT (**A**), and switches its H-bond partner to T62^1.27(NT)^ in the inward-facing conformation (**B**).

A series of previously identified interaction networks [16] that were viewed as “gates”, were found here to participate in the conformational rearrangements underlying the state-to-state transitions by a mechanism of reconfiguration of interaction partners. Thus, networks stabilized by specific interactions such as salt bridges and H-bonds [16] need to be replaced by newly formed interactions to compensate for the energy loss. The intricacy of this reconfiguration suggests that modeling the global configurational changes based on global 3D folding-symmetry considerations cannot offer sufficient insight into the transitions. For example, we had shown that in S1-DAT the cation-pi interaction of Y335^6.68^ with R60^1.25(NT)^ stabilizes a salt bridge between R60^1.25(NT)^ and D436^8.74^, and that this intracellular interaction network regulates conformational transitions in DAT [16]. Here we reported that Y335^6.68^ H-bonds to E428^8.66^ in S1-DAT, but not in the inward-facing conformation (Figure 6) in which Y335^6.68^ forms an H-bond with T62^1.27(NT)^. The loss of the Y335^6.68^ interaction with E428^8.66^ destabilizes S1-DAT and steers the transporter towards an inward-facing conformation. The remodeling of this interaction network alters the capability of the transporter to alternate freely between S1-DAT and the inward-facing conformations that is seen to require a set of local rearrangements, rather than a purely symmetric rearrangement of TM segments.

**Figure 7:**
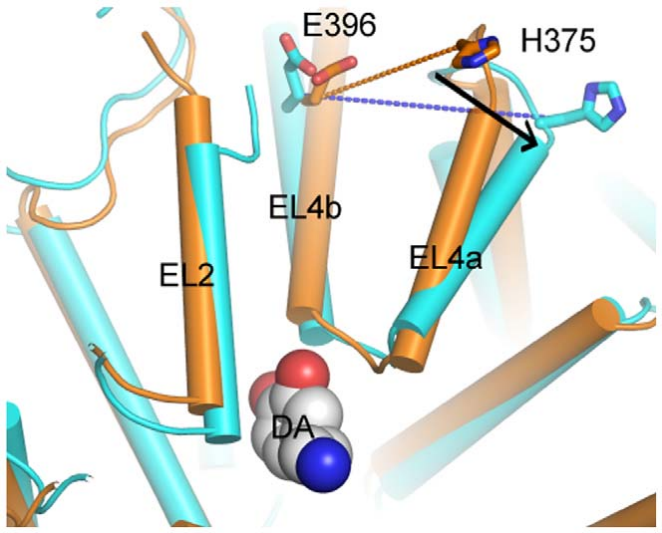
The endogenous Zn^2**+**^ binding site. Extracellular portions of DAT containing the endogenous Zn^2+^ binding site. S1-DAT (orange) and the inward-facing DAT (cyan) are aligned with RMSDTT using the whole structure and rendered in cartoon. The sidechains of Zn^2+^ binding residues H375^EL4a^ and E396^EL4b^ are rendered in sticks. In S1-DAT, the average C_α_ distance between H375^EL4a^ and E396^El4b^ is 13 Å (orange dashed line). The distance increases to 15 Å in the inward-facing conformation (blue dashed line).

Notably, the effect of Y335^6.68^ mutation to Ala was shown to be rescuable by the addition of Zn^2+^ rescues [61], and the mechanism explained by the ability of Zn^2+^ to replace the energetically favorable Y335^6.68^ interaction with R60^(NT)^ thereby reinforcing S1-DAT, and restoring the equilibrium between S1-DAT and the inward-facing conformation that had been lost in the Y335^6.68^A mutant [16]. Zn^2+^ binding to an endogenous site within the extracellular loops of the wild type (WT) DAT was shown to potently inhibit transport, while substrate binding can still take place [3], [62]. Considering the reconfiguration of the interaction network we describe, the details of S1-DAT and the inward-facing conformation we observed provide an atomistic-level mechanism for these findings related to the nature of the endogenous binding site for Zn^2+^ that consists of residues H193^EL2^, H375^EL4a^ and E396^EL4b^. In S1-DAT, the average C_α_ distance between H375^EL4a^ and E396^El4b^ is 13 Å, suitable for Zn^2+^ binding [63] (Figure 7). In contrast, in the inward-facing conformation model this site is no longer suitable for Zn^2+^ binding because both EL2 and EL4b moved down toward S2, and EL4a moved away from EL4b so that the corresponding C_α_ distance increased to 15 Å. Accordingly, Zn^2+^ binding prefers the occluded conformation of WT DAT and by stabilizing it prevents the transition to the inward-open (facing) state, thus inhibiting translocation.

#### The detailed atomistic model of the allosteric mechanism that emerges from this study

Using SMD simulations with extended MD equilibrations we have identified detailed contributions of specific structural elements to the transition between states visited by the transporter molecule in the process of substrate translocation from the primary S1 binding site both to the extracellular and to the intracellular end of the protein. In the movement of substrate from S1 to the intracellular side, these structural elements perform an ordered sequence of local rearrangements that are triggered by the binding of substrate in the S2 site. This allosteric mechanism, identified here for DAT from the SMD simulations and extensive MD equilibrations of the resulting intermediate states, reconfigures a conserved spatial network of interactions (either direct, or through interposed substrate or water molecules) among residues in non-consecutive sequence loci, in a defined temporal sequence. Together, the local conformational changes revealed in the computational modeling of the process give rise to the global rearrangements of TM and loop segments that are captured in the types of conceptual models referring to the molecule undergoing transitions between states, e.g., from outward-open/facing to inward-open/facing states of the transporter.

The detailed atomistic model of the allosteric mechanism that emerges from this study achieves the same conformational endpoints as the classical alternating-access model for transport. However, the classical model is not structurally or mechanistically explicit. It invokes unspecified extracellular and intracellular gates alternating between open and close states without a defined connecting pathway, and would require the transporter molecule to traverse the underlying conformational states sequentially from outward-facing to S1-DAT and then to inward-facing [20], [64]–[66]. Here, we describe for the first time for DAT the molecular details of a substrate movement mechanism in a manner that is directly amenable to experimental verification, as illustrated previously for LeuT [34], [56].

**Figure 8:**
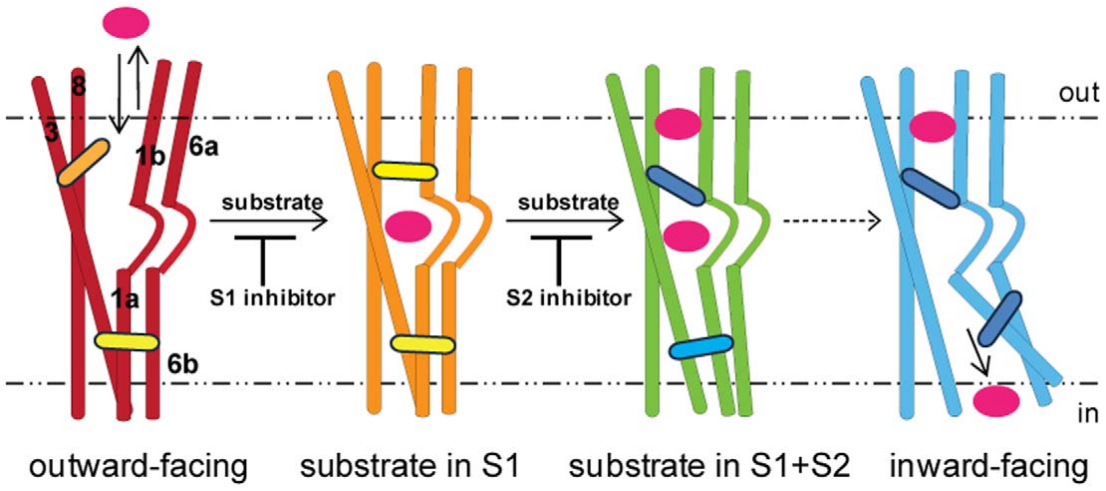
Cartoon model of a substrate translocation cycle for DAT. Substrate binding in the outward-facing model (red) promotes the formation of an occluded conformation (orange). The binding of a second substrate (doubly occupied state in green) induces conformational changes in the S1 site and the intracellular side through conserved interaction networks (colored lozenges) positioned between the S2 and S1 sites, which reorganize the interaction network at the intracellular end, eventually leading to the release of substrate in the S1 site from the inward-facing conformation (cyan). Inhibitors that bind to the S1 site block the formation of S1,S2-DAT and thus the translocation. Inhibitors that bind to the S2 site inhibit the release of S1 and act as translocation de-couplers [29].

The crystal structure of LeuT with a leucine and two Na^+^ bound to the unwound regions of TMs1 and 6 suggested that these unwound regions are relatively flexible and thus may serve as hinges for the conformational transition [15]. Following the classical model, the intracellular TM segments 1a and 6b, and the corresponding extracellular segments 1b and 6a, were proposed to move in an alternating fashion relative to TMs3 and 8 [36]. Our results are in agreement with the identification of these segments as undergoing the most drastic rearrangements in the conformational transition (albeit not to the same extent). However, the nature of the conformational transition suggested by the dynamics revealed in this study is not compatible with simple rigid body movements, especially not for a bundle of TMs [21]. Thus, the allosteric mechanism triggered by the binding of substrate in the S2 site (Figure 8) in the presence of the two Na^+^ and the substrate in the S1 site, which was observed from the atomistic simulations of the DAT structural model, suggests an ordered series of concerted conformational rearrangements in flexible regions that lead to the remodeling of interaction networks in a sequential manner. Specific hinge regions within the TMs (TMs2, 7, 10 and 11) enable the resulting large-scale segment rearrangements that characterize the resulting transition from the occluded to the inward-facing state. The dependence of the large conformational transitions and rearrangements on specific structural elements with identified mechanistic contributions suggests the possibility of various intermediate and functionally-specialized molecular conformations that can be adopted by individual members of the NSS family of transporter proteins.

## Materials and Methods

### Construction of function-related conformational states of DAT

#### DAT residue numbering

The DAT residue numbering scheme used here includes, in addition to the sequence-based numbering, a generic numbering system defined in [35], [67]. According to this scheme, the most conserved residue in each TM is assigned a number 50, and then a pair of numbers (A1.A2) is used to identify each residue, where A1 refers to the TM number and A2 denotes the position of the amino acid relative to the most conserved residue in the TM (A2 numbers decrease from 50 towards the N-terminus and increase towards the C-terminus).

#### Homology modeling of DAT and construction of the simulation system

We had recently described results for a homology model of DAT and simulated it in explicit water and lipid environment [39]. The general protocol and the structure-based sequence alignment for homology modeling and ligand docking is as described there and earlier [35]. Briefly, the homology model uses as the template the known crystal structures for the cognate and homologous structure of LeuT [15]. DA was placed in the S1 site by aligning its amine group and hydrophobic portion with those of the structure leucine in the LeuT structure, and the long equilibration refines the interactions between DA and DAT. The two Na^+^ ions were positioned equivalently to those in LeuT and a Cl^−^ ion was placed based on the chloride binding site described in [42]. The final model was immersed in an explicit water/lipid box to construct the simulated system.

#### Substrate movement towards the extracellular side

Constant velocity SMD simulations were used to explore the extracellular translocation pathway and the S2 site following a protocol described previously for a similar study of LeuT [29]. The SMD simulations were performed on an equilibrated DAT model with a substrate present in the S1 site [39]. A velocity of 4 Å/ns and a harmonic constant of 4 kcal/(mol·Å^2^) were used in the pulling protocol of SMD for the substrate in the S1 site moving towards the extracellular side. As before, the force is applied through a connecting spring tethered at the center of mass of the ligand [29], [68], [69]. As described for the LeuT protocol, about 100 residues in the bottom parts of TMs 2, 4, 5, 7, 9, 10, 11 and 12 were constrained in the Z direction during the SMD. The entire simulation was performed in two phases. In Phase I, 2 ns of SMD simulation was followed by 4 ns of equilibration, while in Phase II 2 ns of SMD simulation was followed by 10 ns of equilibration. After 10 ns of equilibration a substrate was introduced again in the S1 site and the entire system with the two substrates (S1,S2-DAT) was equilibrated for 25 ns.

#### Substrate movement towards the cytoplasm

The SMD simulation was performed on the equilibrated S1,S2-DAT model to explore the intracellular translocation pathway and an inward-facing conformation. Step-wise decreased velocities of 10 Å/ns (for the first 200 ps), 5 Å/ns (for the subsequent 300 ps) and 2.5 Å/ns (for the last 1.5 ns) and a harmonic constant of 4 kcal/(mol·Å2) were used to move the substrate towards the cytoplasm in the established SMD protocol. About 100 residues in the upper part of TMs4, 5, 7, 9, 10, 11 and 12 were constrained in the Z direction during the SMD. Two parallel simulations were carried out to pull substrate from the S1 site to the intracellular side. The first simulation was performed in two phases. In Phase I, 2 ns of SMD simulation was followed by 4 ns of equilibration, while in Phase II 2 ns of SMD simulation was followed by 15 ns of equilibration. The second simulation was performed in three phases. In Phases I and II, 2 ns of SMD simulation was followed by 4 ns of equilibration, while in Phase III 2 ns of SMD simulation was followed by 15 ns of equilibration.

#### Residues in the S1 and S2 sites

Residues were identified as part of the S1 site if during the dynamics simulations they are within 3.5 Å of the substrate at the S1 site for more than 5% of the time during equilibration of either S1-DATor S1,S2-DAT. Residues were treated as part of the S2 site if they are within 3.5 Å of the substrate at the S2 site for more than 5% of the time during equilibration of either S1,S2-DAT or the inward-facing model. For the analysis, the first 5 ns trajectories were discarded. Snapshots were extracted every 10 ps.

#### Identifying residues in the transport pathway

Simuflations moving the substrate towards the extracellular space in the single substrate model were used to identify residues in the transport pathway from the extracellular side to the S1 site of DAT. Residues within 3.5 Å of the substrate were identified at every 2 ps during SMD and at every 5 ps during the equilibration. A complete list of residues that remained in contact with the substrate as it moved from the S1 site towards the extracellular side was prepared for DAT. For every residue in the complete list the time point when a given residue first made contact with the substrate and when it was last in contact with the substrate was recorded for each individual SMD and MD trajectories. For every residue in the list, the percentage of time for which the given residue remained in contact with the substrate between its first and last contact time point was calculated. The residues that remained in contact with the substrate for more than 5% of the time in any individual trajectory were classified as belonging to the extracellular transport pathway. Similarly, simulations of S1,S2-DAT that pulled the substrate towards the cytoplasm were used for identifying the residues in the transport pathway from the S1 site to the cytoplasm of DAT. Residues in either the S1 or S2 site were excluded from transport pathways.

### Structural Analysis

#### Number of waters in DAT along the transport pathway

The internal water pathway in DAT was monitored from the average numbers of water molecules in the pathway along the Z coordinate (the membrane normal) every 50 ps for the last 1 ns of various equilibration trajectories of DAT: S1-DAT, S1,S2-DAT and the inward-facing conformation. All trajectories were aligned to a reference (S1-DAT) before counting.

#### Substrate-water interaction energies

The interaction energies were calculated with the CHARMM27 force field [70] using NAMD [71]. All the water molecules were treated as one group and DA as the other.

#### Calculation of dihedral angles

Dihedral angles (χ1, χ2) in the rotamers of residues F76^1.42^, F332^6.65^ and Y335^6.68^ were calculated with ptraj in AMBER9 [72] every 5 ps for the 25 ns equilibration trajectory of the S1,S2-DAT. Similarly, dihedral angles (χ1, χ2) and ϕ, ψ angles of residues W63^1.29^, F69^1.35^, F76^1.42^, F332^6.65^, Y335^6.68^ and E428^8.66^ were calculated for the final 2 ns equilibration trajectories of S1-DAT, inward-facing and S1,S2-DAT models.

#### Calculation of Solvent accessibility surface area (SASA)

For W63^1.29^, F69^1.35^, F76^1.42^, F332^6.65^, Y335^6.68^ and E428^8.66^ values were calculated from the 25 ns S1,S2-DAT equilibration trajectory and the two intracellular pulling trajectories. SASA was recorded every 5 ps for the equilibration trajectories and every 2 ps for the SMD trajectories. Only surface area accessible to solvent was counted; surface area exposed to lipids was treated as buried. SASA percentage was obtained by dividing the SASA value for residue X by a reference value calculated for X in a Gly-X-Gly tripeptide in extended conformation [73].

#### Calculation of helix kink parameters

To describe local distortions in TMs during the MD simulation caused by proline or consecutive glycine [74] as well as other helix-disrupting residues serine, threonine and cysteine [50], we calculated bend and face shift angles around the following residues: L80^1.46^, P101^2.39^, Pro112^2.50^, S149^3.43^, G153^3.47^, S254^4.61^, T269^5.46^, S354^7.39^, S357^7.42^, S422^8.60^, G426^8.64^, T456^9.49^, G481^10.53^ and S483^10.56^. Calculation was also carried out for Leu80^1.46^ to quantify the changes in TM1a and TM1b since L80^1.46^ is located in the unwound region between TM1a and TM1b. The bend angle, which measures the extent of helical kink, is defined as the angle between pre-kink and post-kink parts in a TM, and the face shift angle describes the distortion that causes a helix to twist in such a way that amino acids previously facing the same side of the helix are now shifted and positioned on different sides of the TM [59].

To quantify the changes in the helix distortion parameters throughout the MD trajectory, we used the ProKink package [59] in the publicly available software Simulaid [60]. Details about the geometric definitions and the computational protocol implemented in ProKink can be found in [59]. In short, DAT snapshots at different time-points were fitted onto the starting reference structure of the protein, and the C_α_ atom of the Pro or other helix-disrupting residue in the relevant TM was positioned at the Cartesian origin. To calculate the bend angle of a helix, the coordinate system was rotated for each trajectory frame around the axis passing through the pre-proline helical segment until the long axes of the post and pre-proline parts were in the same plane. From this orientation, the bend or kink angle was measured as the angle between the axes of the two parts of the helix. To obtain the face shift angle, for each snapshot the post-proline segment, which included the Pro C_α_ atom was rotated so that both pre and post-proline helical parts shared a long axis [59]. The face shift angle was then calculated as the angle between projections of two vectors onto the plane perpendicular to the long axis: these vectors are the on connecting the Pro C_α_ atom with the Cartesian origin, and the average vector connecting the C_α_ atoms of the (*i*-3) and (*i*-4) amino acids with the origin.

#### Aligning different conformational states with RMSDTT to define global movements

Inward-facing conformations were aligned to occluded conformations for both DAT and LeuT using the plugin RMSDTT [53] of the VMD (Visual Molecular Dynamics) [75] program. The iterative fitting implemented in the RMSDTT plugin performs a by-residue weighted pairwise fitting, such that after each iteration, residues with lower average RMSD are assigned higher weights in the next iteration. A similar idea for improving the quality of the commonly used RMSD comparison had been described independently by Damm and Carlson [76]. In total, three iterations of fitting were carried out with default fitting parameters. The reported residue-based RMSD was calculated with RMSDTT when aligning the whole structure.

For additional methods, see Text S1 in File S1.

## Supporting Information

File S1 Supplementary Methods details, figures, and tables detailing structural rearrangements in the different conformational states of DAT.
(DOC)

File S2 Hinge regions underlying the global and local conformational rearrangements in DAT and the hinge regions enabling these changes.
(DOC)
