# Supplementary material for: The Substrate-Driven Transition to an Inward-Facing Conformation in the Functional Mechanism of the Dopamine Transporter
Source: PLoS One. 2011 Jan 27;6(1):e16350. doi: 10.1371/journal.pone.0016350 (PMC3029329; doi:10.1371/journal.pone.0016350)
Supplement: File S1 — Supplementary Methods details, figures, and tables detailing structural rearrangements in the different conformational states of DAT. (DOC) [file pone.0016350.s001.doc]

# The Substrate-driven Transition to an Inward-facing Conformation in the Functional Mechanism of the Dopamine Transporter

Jufang Shan, Jonathan A Javitch, Lei Shi and Harel Weinstein

# Figure S1. Conformational changes in S1,S2-DAT compared to S1-DAT.


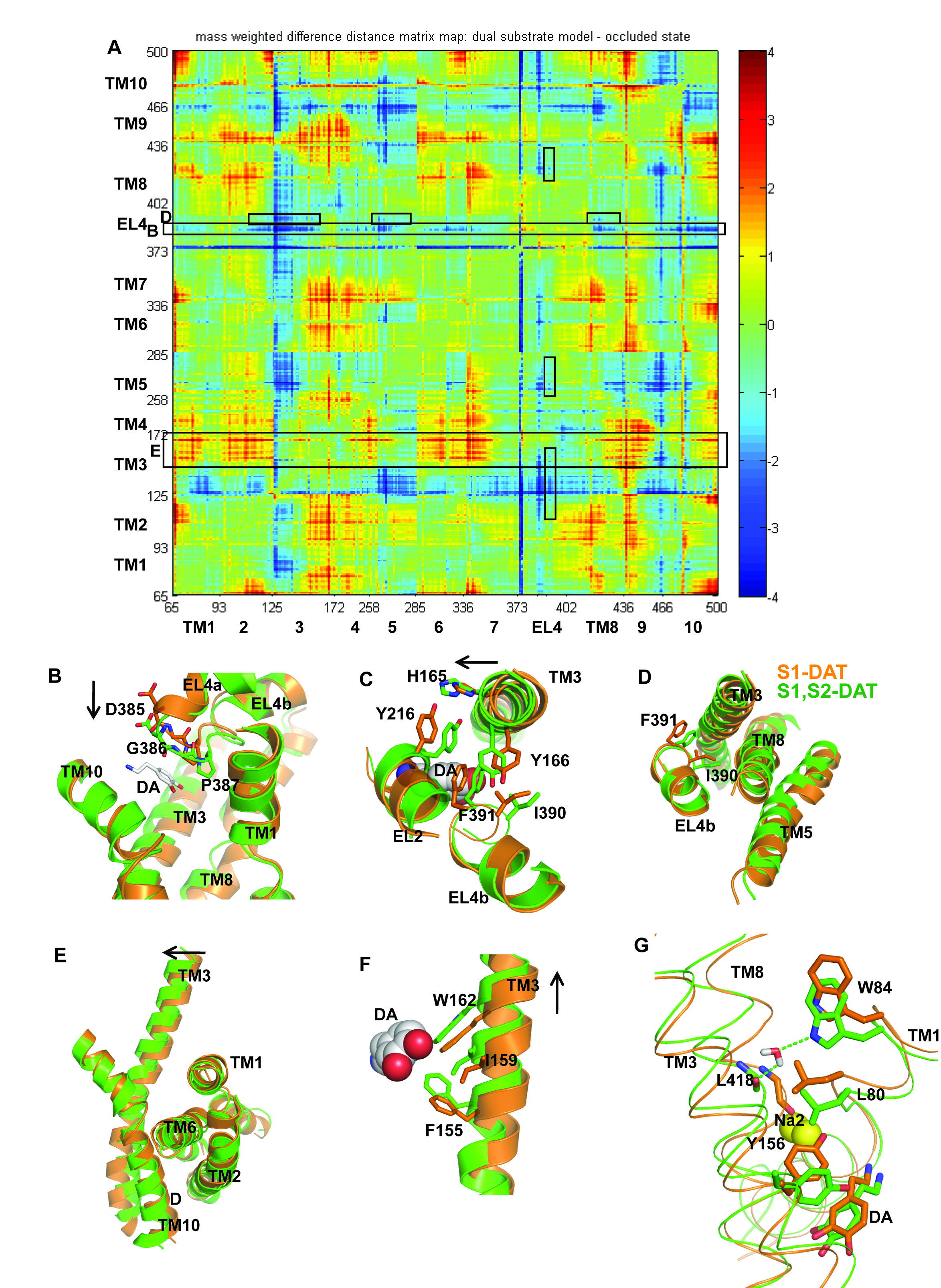


(**A**) The mass-weighted average difference distance matrix map (DDMP) for TM1 to TM10 and EL4 of S1,S2-DAT and S1-DAT. The distance matrices for S1-DAT and for S1,S2-DAT were calculated with ptraj [1], and then the S1-DAT matrix was subtracted from the S1,S2-DAT resulting in the DDMP. The DDMP was plotted with Matlab 2007b (The MathWorks Inc.) and colored by difference distance as shown in the color bar: distance changes > 4 Å are in red, equal to 0 Å in green, and < -4 Å, in blue. Positive change values indicate residues that moved away from each other when S1-DAT transitioned to S1,S2-DAT; negative changes mean that residues moved closer to each other in the transition; zero changes indicate that residues either didn’t move or moved together in the same direction.
(**B**) D385^EL4^, G386^EL4^ and P387^EL4^ moved downwards as the distances between them, and the intracellular segment of TM1 to TM10 in S1,S2-DAT, became smaller (highlighted in Figure S1A).
(**C**) When moving down, EL2 and EL4 pushed the extracellular segment of TM3 inwards to the S2 site through interactions between Y216^EL2^, I390^EL4^ and F391^EL4^ on the two loops, and H165^3.59^ and Y166^3.60^ on TM3.
(**D**) In S1,S2-DAT, residues I390^EL4^ and F391^EL4^ moved closer to TMs3, 5 and the middle part of TM8.
(**E**) The extracellular segment of TM3 moved inward and away from several TMs including those that are opposite to TM3: TMs1a, 2, 6 and the extracellular segment of TM10.
(**F**) TM3 shifted up slightly along its helix axis and three residues: F155^3.49^, I159^3.53^ and W162^3.56^ were positioned for interaction with S2.
(**G**) In the S1,S2-DAT, L418^8.56^ flips its carbonyl group to interact through a water molecule with the downward moved W84^1.50^. The middle portion of TM8 moves away from TM1. In S1-DAT, L418^8.56^ coordinates with Na2. Colors are the same as in Figure 3. Helices are represented in cartoon, residues are in sticks and DA is either in sticks (**B**) or spheres (**C**, **F**).

**Figure S2. SASA for the aromatic cluster residues during equilibration of S1,S2-DAT.**


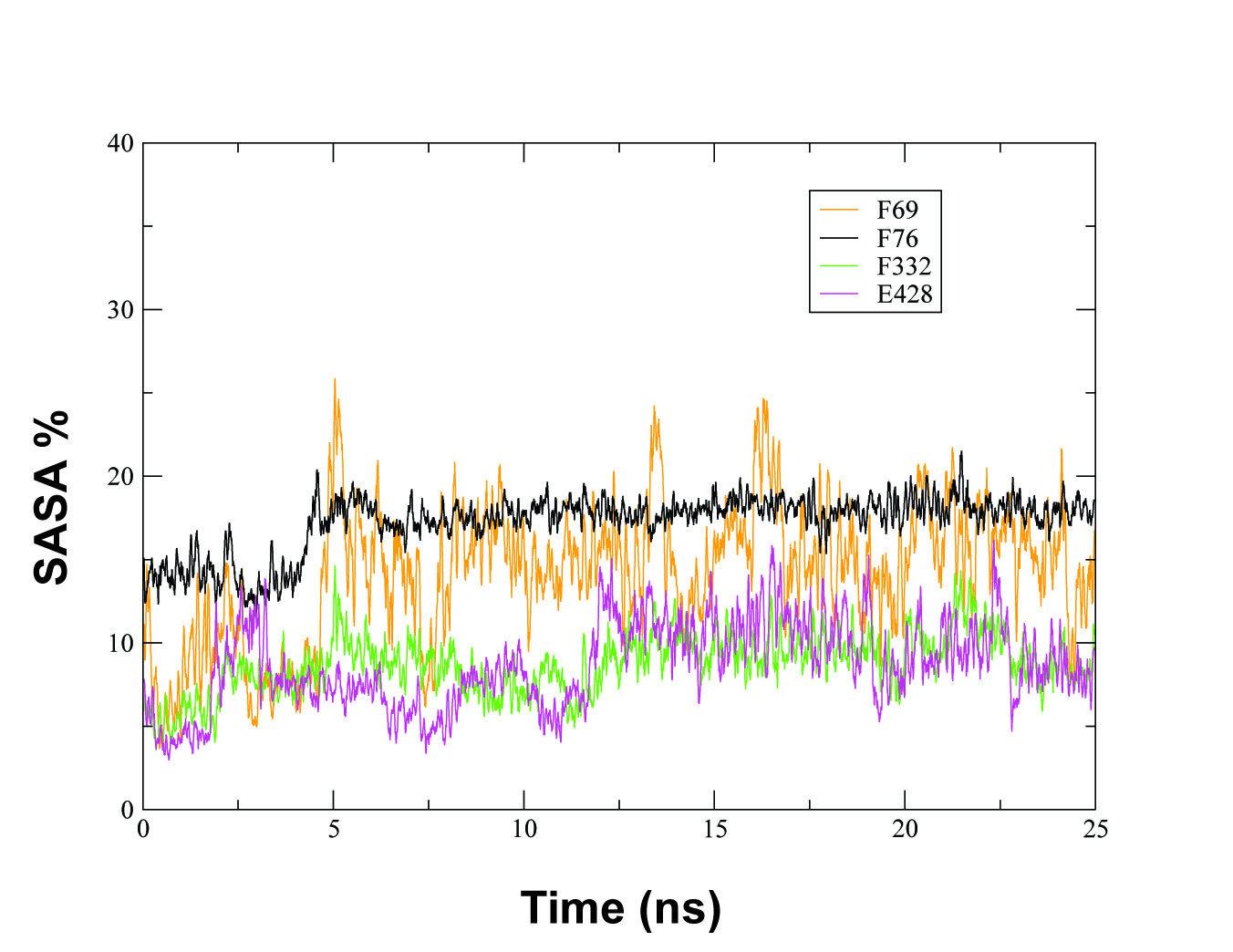


**Figure S3. Spearman’s rank test on dihedrals of the aromatic cluster residues during intracellular pulling.**

**
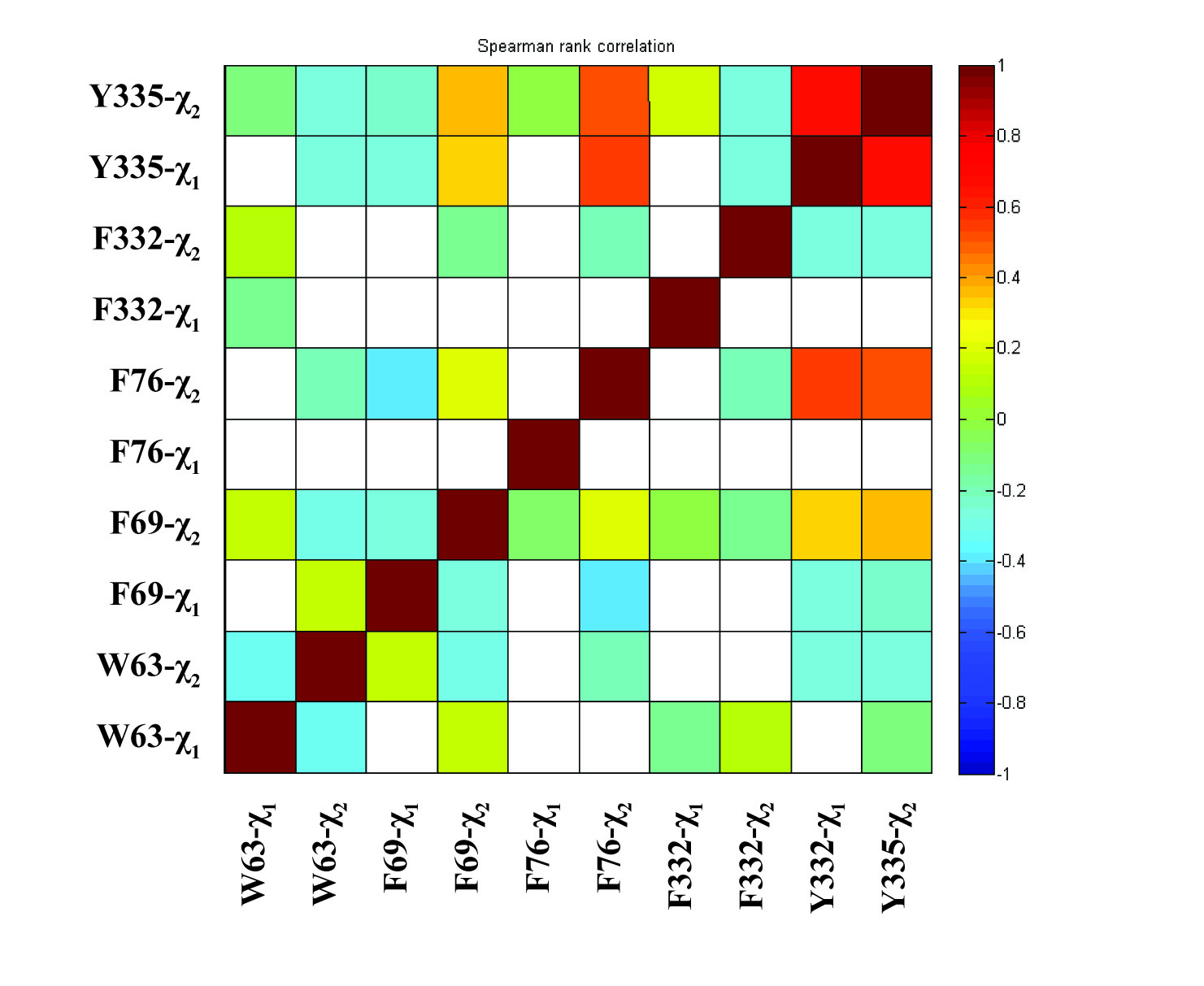
**

White indicates a P-value greater than 0.0001 (not significant).

**Figure S4. Residue-based RMSD of the inward-facing conformation to S1-DAT by RMSDTT.**

**
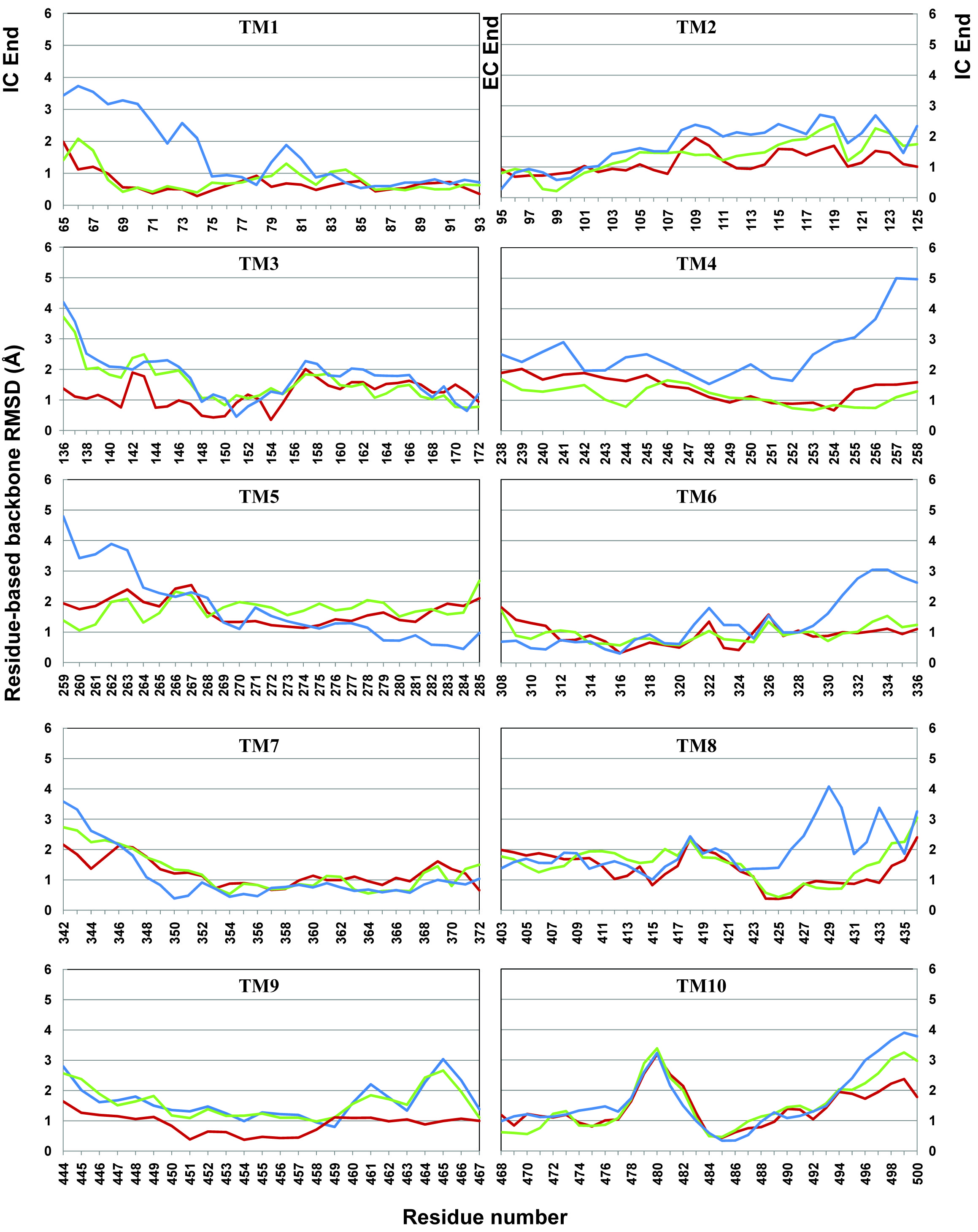
**

The model of DAT after substrate has moved from the S1 site to the extracellular side (shown in red); the S1,S2-DAT construct (in blue), and the inward-facing conformation (green) were aligned to S1-DAT using RMSDTT [2] and RMSD values were plotted for residues in TM1 to TM10.

**Table S1. Rotamer changes from S1-DAT to S1,S2-DAT.**

| Residue | Dihedrals | S1-DAT *^a^* | | | | S1,S2-DAT *^b^* | | | |
| --- | --- | --- | --- | --- | --- | --- | --- | --- | --- |
|  |  | Average *^c^* | trans % *^d^* | g+ % *^d^* | g- % *^d^* | Average *^c^* | trans % *^d^* | g+ % *^d^* | g- % *^d^* |
| F76^1.42^ | φ | -59.7 |  |  |  | -68.6 |  |  |  |
| L80^1.46^ | φ | -75.6 |  |  |  | -60.6 |  |  |  |
|  | ψ | -27.9 |  |  |  | -34.8 |  |  |  |
|  | χ_2_ | n/a | 95.4 | 1.5 | 0 | n/a | 83.6 | 4.3 | 0.7 |
| W84^1.50^ | ψ | -47.3 |  |  |  | -52.5 |  |  |  |
| F155^3.49^ | φ | -64.3 |  |  |  | -72.5 |  |  |  |
|  | ψ | -44.1 |  |  |  | -34.1 |  |  |  |
|  | χ_1_ | 188.9 |  | 0 | 0 |  | 35.2 | 54.5 | 0 |
|  | χ_2_ | 44.0 |  |  |  | 30.4 |  |  |  |
| Y156^3.50^ | χ_1_ | -137.2 |  |  |  | -66.5 |  |  |  |
|  | χ_2_ | 104.2 |  |  |  | 138.1 |  |  |  |
| S262^5.40^ | χ_1_ | n/a | 55.1 | 0 | 40 |  | 66.4 | 24.5 | 0 |
| F320^6.53^ | χ_2_ | 66.4 |  |  |  | 14.5 |  |  |  |
| F332^6.65^ | χ_1_ | -75.5 |  |  |  | -82.6 |  |  |  |
|  | χ_2_ | -171.8 |  |  |  | -165.0 |  |  |  |
| L418^8.56^ | φ | -69.3 |  |  |  | -76.7 |  |  |  |
|  | ψ | -18.0 |  |  |  | 112.6 |  |  |  |
|  | χ_1_ | -55.3 | 0 | 0 | 96.5 | n/a | 85.3 | 0 | 11.3 |
|  | χ_2_ | 181.6 | 98.3 | 0 | 0 | n/a | 7.9 | 88.2 | 0 |
| M424^8.62^ | χ_1_ | n/a | 61.6 | 3.7 | 20.4 | n/a | 35.6 | 0 | 52.8 |
|  | χ_2_ | n/a | 62.1 | 16.4 | 6.4 | n/a | 89.2 | 1.2 | 0 |

*^a,b^* Dihedral angles were calculated using the last 5 ns of equilibration trajectories of S1-DAT (the 12^th^ – 16^th^ ns) *^a^* and S1,S2-DAT (the 21^st^ – 25^th^ ns) *^b^*, respectively. Snapshots were extracted every 5 ps.
*^c,d^* If the dihedrals are continuous in the 5 ns simulation, averages are calculated; otherwise, the percentage of trans, g-, g+ *^d^* were reported, which are defined as within ±25 degrees of 180, -60 and +60 degrees, respectively.

**Table S2. Dihedrals of the aromatic cluster in different conformational states of DAT.**

| Residues | Dihedrals | S1-DAT *^a^* | S1,S2-DAT *^b^* | Inward-facing *^c^* |
| --- | --- | --- | --- | --- |
| W63^1.29^ | φ | -69.4 | -75.1* | -89.3* |
|  | ψ | -56.9 | -89.2* | -91.1* |
|  | χ_1_ | -58.9 | -69.0* | -57.8 |
|  | χ_2_ | 82.7 | 100.7* | 111.2* |
| F69^1.35^ | φ | -69.5 | -70.6 | -78.5* |
|  | ψ | -44.6 | -49.2 | -54.0* |
|  | χ_1_ | 185.0 | 182.3 | 188.7 |
|  | χ_2_ | 62.7 | 54.9* | 235.1* |
| F76^1.42^ | φ | -59.7 | -69.6* | -82.0* |
|  | ψ | -44.5 | -47.0 | -45.1 |
|  | χ_1_ | 181.9 | 176.9 | 184.2 |
|  | χ_2_ | 71.2 | 73.5 | 75.7 |
| F332^6.65^ | φ | -61.8 | -62.9 | -62.1 |
|  | ψ | -48.4 | -47.8 | -47.3 |
|  | χ_1_ | -75.7 | -82.6* | 193.5* |
|  | χ_2_ | -171.8 | -165.0* | 83.5* |
| Y335^6.68^ | φ | -74.2 | -76.8 | -75.9 |
|  | ψ | -21.6 | -6.67* | -18.4 |
|  | χ_1_ | -69.9 | -75.2* | 193.7* |
|  | χ_2_ | 104.9 | 117.3* | -86.8*^d^ |
| E428^8.66^ | φ | -58.8 | -61.9 | -64.2* |
|  | ψ | -44.8 | -40.5 | -47.8 |
|  | χ_1_ | 182.6 | 184.5 | 182.8 |
|  | χ_2_ | 64.1 | 58.9* | 177.7* |

*^a,b,c^* Dihedral angles were calculated using the last 5 ns of equilibration trajectories of *^a^* S1-DAT (the 12^th^ – 16^th^ ns), *^b^* S1,S2-DAT (the 21^st^ – 25^th^ ns), and *^c^* the inward-facing conformation (the 11^th^ – 15^th^ ns), respectively. Dihedral were calculated on snapshots extracted every 5 ps using ptraj [30]. Average values were reported.
*^*^* changes more than 5 degrees compared to S1-DAT.
*^d^* Tyr335^6.88^-χ_2_ was discontinuous with 8.2% trans, 8.2% g- , and 8.7% g+. 8.2%, g+ 8.7%. Trans, g-, g+ were defined as within ±25 degrees of 180, -60 and +60 degrees, respectively.

**Table S3. RMSD for the extracellular and intracellular segments when individual TM was fitted with its extracellular segment.**

|  | Extracellular Segments | | Intracellular Segments | |
| --- | --- | --- | --- | --- |
|  | Residues | RMSD (Å) | Residues | RMSD (Å) |
| TM1 | L80^1.46^ – N93^1.59^ | 0.4 | K65^1.31^ – D79^1.45^ | 4.4 |
| TM2 | G95^2.33^ – P101^2.39^ | 0.7 | Y102^2.40^ – R125^2.63^ | 6.3 |
|  | Y102^2.40^ – M111^2.49^ | 0.5 | P112^2.50^ – R125^2.63^ | 2.9 |
| TM3 | F154^3.48^ – T172^3.66^ | 0.6 | P136^3.30^ – G153^3.47^ | 3.1 |
| TM4 | W238^4.45^ – F253^4.60^ | 0.7 | S254^4.61^ – G258^4.65^ | 3.8 |
| TM5 | A270^5.47^ – V285^5.62^ | 0.5 | V259^5.35^ – T269^5.46^ | 3.2 |
| TM6 | A308^6.41^ – L322^6.55^ | 0.4 | V328^6.61^ – N336^6.69^ | 2.6 |
| TM7 | S354^7.39^ – A373^7.58^ | 0.7 | M342^7.26^ – S354^7.39^ | 1.2 |
| TM8 | L403^8.37^ – D421^8.59^ | 0.9 | S422^8.60^ – D436^8.74^ | 3.7 |
| TM9 | T456^9.49^ – N466^9.59^ | 0.5 | H444^9.37^ – A455^9.48^ | 1.1 |
| TM10 | G468^10.40^ – F478^10.50^ | 0.6 | A479^10.51^ – G500^10.73^ | 3.4 |
| TM11 | S528^11.49^ – F543^11.64^ | 0.5 | L518^11.39^ – V527^11.48^ | 1.2 |
| TM12 | D555^12.31^ – S568^12.45^ | 0.6 | M569^12.46^ – G585^12.62^ | 3.3 |

**Text S1. Supporting** **Methods**

***Spearman’s’ Test*** *–* Spearman’s test was carried out using R.

***Calculation of difference distance matrix map (DDMP)*** *–* The mass-weighted distance matrix was calculated with ptraj in AMBER9 [1] on snapshots saved every 5 ps during the last 5 ns of equilibration trajectories of S1-DAT and S1,S2-DAT, respectively. The DDMP was generated by subtracting distances in S1-DAT from S1,S2-DAT, and plotted with Matlab 2007b (The MathWorks, Inc.).

***Individual TM alignments using their extracellular segments to identify local TM distortions*** *–* The local conformational changes in TM11 and TM2 were not addressed in this study due to the poor homology between LeuT and DAT. In addition, TM11 and TM12 do not belong to the 10-TM functional core of NSSs. Except for TM11 and TM12, each TM in the inward-facing conformation of DAT was superimposed individually on the corresponding TM in S1-DAT using backbone atoms of residues in the extracellular segment. The extracellular segments of TMs were defined arbitrarily based on their residue-based RMSD from global alignment as follows: TM1, L80^1.46^ –N93^1.59^; TM2, G95^2.33^ - P101^2.39^; TM3, F154^3.48^ – T172^3.66^; TM4, W238^4.45^ – F253^4.60^; TM5, A270^5.47^ – V285^5.62^; TM6, A308^6.41^ – L322^6.55^; TM7, S354^7.39^ – A373^7.58^; TM8, L403^8.37^ – D421^8.59^; TM9, T456^9.49^ – N466^9.59^; TM10, G468^10.40^ – F478^10.50^; TM11, S528^11.49^ – F543^11.64^; and TM12, D555^12.31^ – S568^12.45^. No local conformational changes were identified in TM7 and TM9.

**References**

1. Case DA, Darden, T.A., Cheatham, III, T.E., Simmerling, C.L. , Wang, J., et al. (2006) AMBER 9. University of California, San Francisco.
