# Supplementary material for: The Substrate-Driven Transition to an Inward-Facing Conformation in the Functional Mechanism of the Dopamine Transporter
Source: PLoS One. 2011 Jan 27;6(1):e16350. doi: 10.1371/journal.pone.0016350 (PMC3029329; doi:10.1371/journal.pone.0016350)
Supplement: File S2 — Hinge regions underlying the global and local conformational rearrangements in DAT and the hinge regions enabling these changes. (DOC) [file pone.0016350.s002.doc]

# The Substrate-driven Transition to an Inward-facing Conformation in the Functional Mechanism of the Dopamine Transporter

Jufang Shan, Jonathan A Javitch, Lei Shi and Harel Weinstein

# Text S2. Hinge regions underlie local and global conformational changes

#### TM1

The unwound regions in TMs1 and 6 had been postulated to be ligand-related hinges, and TM1a and TM1b have been proposed to move during the conformational transition associated with the translocation cycle [1]. In DAT, the unwound region in TM1 consists of two residues V78^1.44^ and D79^1.45^, and divides TM1 into two helical segments TM1a and TM1b. This unwound region contributes to the S1 site and coordinates both bound Na^+^ ions: D79^1.45^ forms a salt bridge with the protonated amine of DA and coordinates Na1, and the backbone carbonyl of V78^1.44^ coordinates Na2. In the globally aligned inward-facing DAT, TM1a exhibits a significant rearrangement that registers a large residue-based RMSDs of nearly 4 Å relative to S1-DAT. Notably, away from the N-terminus the residue-based RMSD decreases to 1 Å at position G75^1.41^ (a half-turn before the unwound region) and remains close to 1Å for residues in TM1b (Figure S4 in File S1). Therefore, in the globally aligned models, the cytoplasmic TM1a in the inward-facing conformation is seen to be shifted outwards with the unwound region serving as a hinge (Figure S5A in File S2).

To examine whether the conformational change in TM1 was caused by local change in TM1 itself or by the global alignment method, we fitted the backbones of the immobile extracellular section from L80^1.46^ to N93^1.59^ in the inward-facing conformation to those of S1-DAT. A small RMS of 0.4 Å is found this way for the fitted extracellular section, whereas the intracellular section registers a big RMS of 4.4 Å (Table S3 in File S1). The conformational change observed in this way (Figure S5B in File S2) is similar to the change seen when the two conformations were aligned using the whole protein with the RMSDTT method [2].

To quantify further the structural change in TM1, the helix kink was quantified with a ProKink calculation performed at position L80^1.46^, after the unwound region. Compared to S1-DAT the bend angle of this helix in the inward-facing conformation changed by 6 degrees, and the face shift altered by 40 degrees (Table 5). Residues around the unwound region are important for the translocation cycle even if they are not directly involved in binding S1 and Na^+^, as evidenced by the findings that mutations G94^1.41^C, A96^1.43^C and L99^1.46^C in hSERT [3], and G59^1.41^C, I62^1.44^C and L64^1.46^C in GAT-1, decrease substrate uptake [4]. All these functionally important positions 1.41, 1.43, 1.44 and 1.46 are highly conserved in NSSs [5].

#### TM2

The two highly conserved [5] and functionally important prolines in TM2 are P101^2.39^ and P112^2.50^. P101^2.39^A mutation in rDAT decreases DA uptake affinity and the V_max_ of DA transport [6,7], and increases efflux [8]. The P101^2.39^C mutation in hDAT decreases K_m_ for tyramine uptake [9]; P97^2.39^C in hNET decreases apparent affinity for cocaine, and increases K_D_ and B_max_ of ^3^H-nisoxetine [10]. P112^2.50^A in rDAT decreases DA uptake affinity and the V_max_ of DA transport [6,7]. The mutants P112^2.50^C in DAT [9], and P131^2.50^C in rSERT are completely inactive [11]; P108^2.50^C in hNET decreases uptake of ^3^H-noradrenaline and ^3^H-nisoxetine binding [10]. Considering the functional impact of these mutations and the kinks these two prolines introduced in TM2, we proposed earlier that a pincer-like configuration in TM2 and the flexibility introduced by P101^2.39^ and P112^2.50^ are important to the interconversion between S1-DAT and the inward-facing conformation [9]. Indeed, we find here that these two prolines change the direction of the helix axis of TM2 twice, separating TM2 into three segments: the extracellular one N-terminal to P101^2.39^, the one between these two prolines, and the intracellular one C-terminal to P112^2.50^. These three segments exhibited different global conformational rearrangements when DAT transited from S1-DAT to the inward-facing conformation (Figure S5A, C, D in File S2). In particular, the extracellular segment containing the glycine at the N-terminus of TM2 moved towards EL3, whereas the middle segment tilted outwards together with the intracellular segment. Fitting TM2 using either the first or second helical segment also showed that TM2 rearranged and distortions were observed around the two prolines. Indeed, the bend angles at P101^2.39^ and P112^2.50^ changed significantly in the transition from S1-DAT to the inward-facing conformation, by 7 and 5 degrees, respectively (Table 5).

The global conformational change in the N-terminus of TM2 is associated with that of the EL1 loop. The highly conserved NGGGAF motif in EL1-TM2 moved towards EL3, a change that is consistent with findings in the literature. Several mutational studies suggest that EL1 undergoes conformational change during translocation cycle in GLYT2a [12] and SERT [11], and is required to maintain transport activity in GAT-1 [13] and rSERT [14]. In addition, Yamashita et al. proposed that in the conformational transition during the translocation cycle, the movement in TM1 may be accompanied by a conformational change in the linker between TM1 and TM2 [1]. Indeed, the NGGGAF motif in LeuT moved towards EL3 when transiting from the occluded conformation to an open-to-out conformation, TM1b and TM2 swings outwards and there is local conformational change in the NGGGAF motif [15].

#### TM3

TM3 is involved in ligand binding in both S1 (S149^3.43^, V152^3.46^, T156^3.50^) and S2 (F155^3.49^, I159^3.53^, W162^3.56^), and also lines the extracellular translocation pathway (G153^3.47^, N157^3.51^, I160^3.54^, A163^3.57^). In the globally alignment of the inward-facing conformation to S1-DAT, the extracellular segment of TM3 appears to have moved slightly inwards due to interactions with S2 (Figure 5A in File S2), whereas its intracellular segment (together with that of TM12) moved inwards to remain associated with the rest of the transporter (Figure S6 in File S2). The intracellular segment of TM3 moved in somewhat toward the place originally occupied by TM6, but the H-bond between S149^3.43^ and the carbonyl of F326^6.59^ was maintained during the transition. In contrast, due to rotamer changes in Y151^3.45^, the H-bond between Y151^3.45^ and A565^12.42^ broke although TM3 and TM12 moved inwards together (Figure S6A, B in File S2).

Residue-based RMSDs calculated with RMSDTT for the alignment of the two conformations identifies a hinge region in the middle of TM3 (Figure S4 in File S1). This was further confirmed by aligning the extracellular segment of TM3, which shows that the intracellular segment underwent rearrangement with an RMSD of 3.1 Å whereas the extracellular segments are only 0.6 Å apart by RMSD (Table S3 in File S1). Here, the hinge region contains two helix-disrupting residues S149^3.43^ and G153^3.47^. The bend angles of TM3 changed significantly at these two residues during the conformational transition, by 8 and 9 degrees, respectively (Table 5). Mutation studies suggest that this hinge region is functionally important, as the V141^3.43^C, Y143^3.45^C and N145^3.47^C mutations in the creatine transporter reduce uptake [16] and W135^3.45^S in mouse GAT reduces GABA transport [17]. Considering the structural flexibility and functional importance of this region, we hypothesize that this hinge region is involved in the conformational transition associated with the translocation cycle.

#### TMs 4 and 5

TMs 4 and 5 are at the outer rim of the transmembrane domain. They form a V-shaped structure wrapping around TMs3 and 8. During intracellular pulling of DA in the SMD simulation, the ligand exits from a channel formed by TMs 1, 5, 6 and 8 (Figure 5B in File S2) when TMs1, 5 and 8 move outwards; TM6 also moves outwards but in an opposite direction, to open the channel. TM4, whose intracellular segment neighbors that of TM8, also moved outwards and also shifted up along the helix axis about one residue (~1/4 turn). The upwards shift explains the relatively large residue-based RMSD of the extracellular segment (~2–3 Å) compared to other extracellular segments (Figure S4 in File S1). Fitting the extracellular segment of TM4 clearly showed that residue S254^4.61^ was at the hinge region of TM4 (Figure S5E in File S2). The bend angle of TM4 changed 12 degrees at this functionally important position (Table 5). S253^4.61^A in rDAT [18] and C228^4.61^S in GAT-1 [19] reduce transport activity; S253^4.61^A together with Y251^4.59^A in rDAT also reduces the affinity for CFT but increases the affinity for DA [20].

In the superposition, we observed large residue-based RMSDs for the intracellular portion of TM5 (up to 6 Å) but only around 1 Å for the extracellular segment (Figure S4 in File S1). Aligning the extracellular portion of TM5 in the inward-facing state to that in S1-DAT, showed the global and local rearrangement (Figure S5F in File S2) to have similar patterns, confirming a local distortion in the middle of TM5. A helix-disrupting residue T269^5.46^ played a role in the rearrangement of TM5, and ProKink analysis showed that the bend angle at T269^5.46^ changed by 10 degrees in the transition from S1-DAT to the inward-facing conformation. Notably, two conserved helix-disrupting residues, T271^5.48^ and P273^5.50^, are located half-turn and one turn after T269^5.46^, respectively, and might coordinate T269^5.46^ in the rearrangement of TM5. The coordination between threonine and proline has been reported before in a functional important T*X*P motif that introduces a bend in TM2 of CCR5 [21]. Interestingly, the three residues T269^5.46^, T271^5.48^ and P273^5.50^ were found to be important for the translocation cycle in studies showing that both T284^5.46^C and T286^5.48^C in SERT decrease K_m_ and increase V_max_ for 5-HT transport [22]. T268^5.48^A in hNET affects the affinities for different ligands differently [23]; P273^5.50^A in rDAT reduces the K_m_ values of DA efflux and the apparent velocities of DA efflux [7], the V_max_ of DA uptake, and the affinities for both DA and WIN 35428 binding [6]. Although functionally important, positions 5.46 and 5.50 are not conserved between prokaryotic and eukaryotic NSSs. In LeuT, 5.46 is an alanine and 5.50 is a leucine. However the structural role of these two residues might be mimicked by Pro200^5.48^ in LeuT, which could give LeuT the structural flexibility it needs during the conformational transition we studied.

#### TM6

Like in TM1, the unwound region (G323^6.56^–V324^6.57^–G325^6.58^–F326^6.59^–G327^6.60^) in TM6 participates in S1 ligand binding, with F326^6.59^ in direct contact with DA. The nature of the conformational rearrangement in TM6 associated with the transition from S1-DAT to the inward-facing conformation is also similar to that in TM1: not much movement of the extracellular segment and the unwound region, but more in the intracellular segment TM6b (Figure S7 in File S2), with residue-based RMSDs going up to 3 Å in TM6b (Figure S4 in File S1). Superposition of the TM6a segments (A308^6.41^ – L322^6.55^) of the two conformations reveals a small backbone RMS of 0.6 Å, but a bigger one for TM6b (V328^6.61^ – Y335^6.68^), 2.6 Å (Figure S7 in File S2). The C_α_ atom of Y335^6.68^ in the inward-facing conformation shifted outwards by 3.1 Å from that of S1-DAT, breaking the H-bond between the backbone carbonyl of G327^6.60^ and the sidechain hydroxyl of S429^8.67^ broke (Figure S7A,B in File S2). The movement of TM6b away from TM8, TM4 and TM5 adds to the opening of the channel in the center of the intracellular portion of DAT, allowing DA to exit (Figure S7B in File S2). This is consonant with the observation that residues in the unwound region are functionally important even if they do not bind the ligand in S1: G323^6.56^V in hDAT completely blocks tyramine uptake; P339^6.57^L in hSERT decreases transport activity [24]; L298^6.57^C in rGAT reduces GABA uptake [25]; and G327^6.60^V in hDAT greatly decreases plasma membrane expression and completely blocks substrate transport [26].

#### TM8

TM8 is one of the two TMs that participate in ligand binding in S1 and in lining both the extracellular and intracellular translocation pathways; the other one is TM1. TM8 is also one of the few TMs whose extracellular segments were found to undergo global conformational changes during the transition (Figure 5A in File S2). While its extracellular portion bent outwards, its intracellular portion also bent outwards towards TM4, 5 and 9 (Figure 5B in File S2). As a result, both the extracellular and cytoplasmic ends of TM8 have large residue-based RMSDs of around 3 Å (Figure S4 in File S1) and a hinge region at the middle of the helix. The hinge region contains a S1 binding residue, S422^8.60^, which H-bonds one of the hydroxyl groups in DA. ProKink analysis shows that the face shift in TM8 changed by 44 degrees at S422^8.60^ (Table 5). Considering that S422^8.60^ is involved in S1 ligand binding and conformational transition, it is not surprising that mutating this serine to alanine decreases DA uptake [18], and mutating to threonine or cysteine in GAT-1 increases K_m_ for GABA transport and leads to lithium leak currents [27].

#### TM10

In the TM10 of DAT, there is a “melted” region consisting of the four residues A480^10.52^–G481^10.53^–T482^10.55^–S483^10.56^. This region is not conserved [5], and only one out of the four positions, 10.53, is conserved with helix-disrupting residues Gly/Pro/Thr/Ser/Cys. The other three positions have deletion mutations, e.g., position 10.54 in DAT and positions 10.55 and 10.56 in Tyt1 and TnaT. The extracellular segment N-terminal to, and the intracellular segment C-terminal to this melted region exhibited different global rearrangement when DAT converted from S1-DAT to inward-facing conformations (Figure S5G in File S2). In the extracellular portion one small movement were observed, with residue-based RMSDs of about 1 Å, whereas the intracellular one moved significantly outwards with RMSDs of up to 4 Å (Figure S4 in File S1). The melted region itself also had large RMSDs, likely caused by the lack of secondary structure. Fitting the extracellular segment of TM10 in the two conformations illustrates the TM rearrangement (Figure S5G in File S2). The significant movement in the intracellular TM segment was generated by changes in bend angle at G481^10.53^ (from 32 to 37 degree) and the face shift at S483^10.56^ (from 137 to 81 degree). Despite the lack of conservation, this melted region is functional important as shown by findings that mutation of both 10.55 and 10.56 in rDAT [20], and mutation of 10.56 in hSERT [28,29] affect ligand binding as well as substrate uptake.

**Figure S5. Global and local conformational changes of individual TMs.**


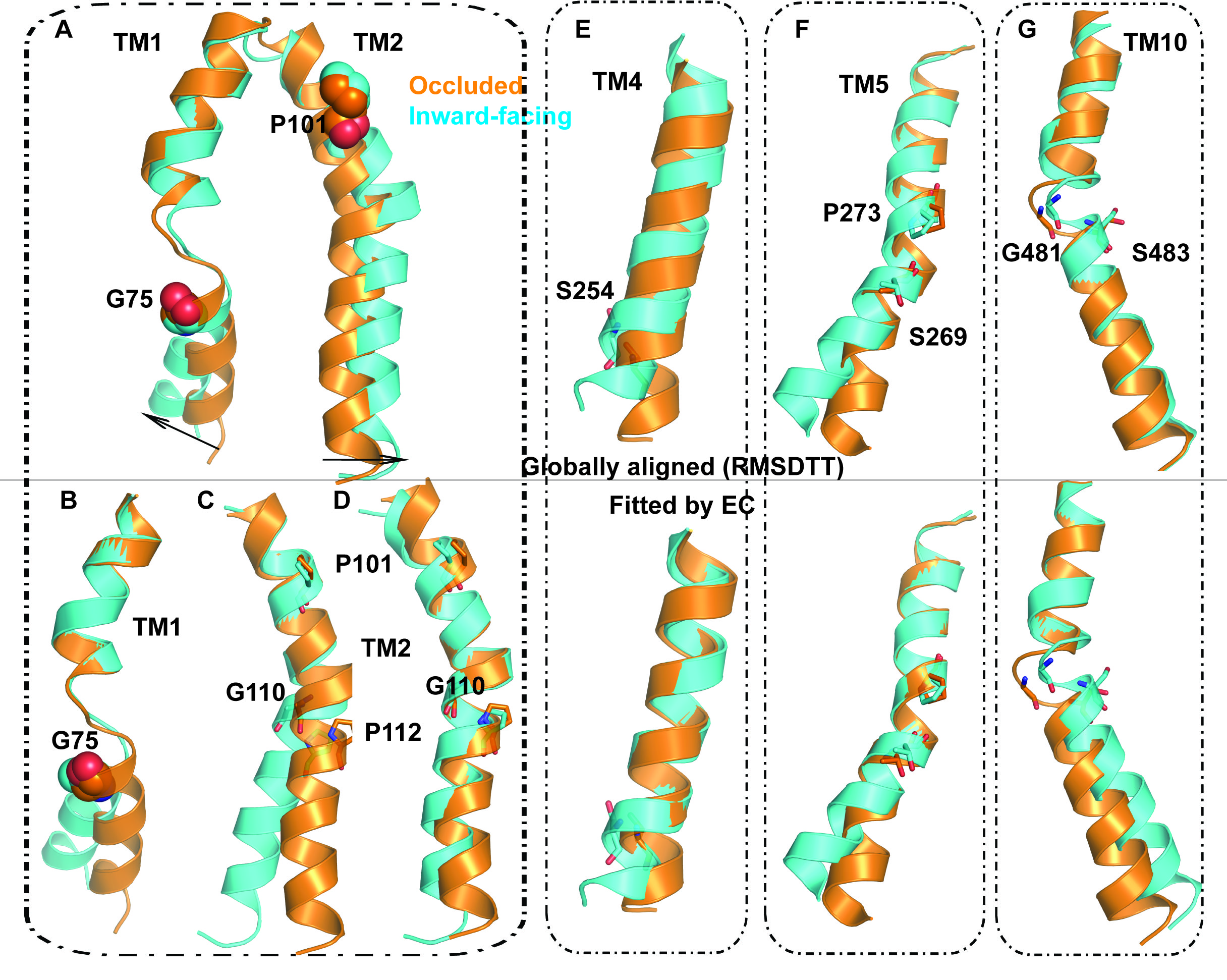


Top panels show global conformational changes from S1-DAT (orange) to the inward facing conformation (cyan) (whole structures were aligned using RMSDTT [2]; the bottom panels show local conformational changes (individual EC segments are aligned as detailed below). (**A**) Global conformational changes in TMs1 and 2. The rearrangements in the EC segments are small while the IC segments starting from the hinge residues (G75^1.41^ and P101^2.39^, in spheres) underwent larger-scale movements.
(**B**–**D**) Local conformational changes in TM1 (**B**) and TM2 (**C**,**D**). TM1 in the inward-facing conformation was fitted to TM1 in S1-DAT using the extracellular segment L80^1.46^ – N93^1.59^. TM2 was fitted to its counterpart using the extracellular segment G95^2.33^ – P101^2.39^ (**C**) or the middle segment Y102^2.40^ – M111^2.49^ (**D**).
(**E**–**G**) Conformational changes in TM4 (**E**), TM5 (**F**) and TM10 (**G**). In bottom panels, individual EC segments were fitted using the following residues: TM4, W238^4.45^ – F253^4.60^; TM5, A270^5.47^ – V285^5.62^; and TM10, G468^10.40^ – F478^10.50^. Hinge residues G75^1.41^, P101^2.39^, G110^2.48^ and P112^2.50^, S254^4.61^, S269^5.46^ and the unwound region G481^10.53^ and T483^10.56^ are shown in spheres or sticks.

**Figure S6. Conformational changes associated with the intracellular segment of TM3.**


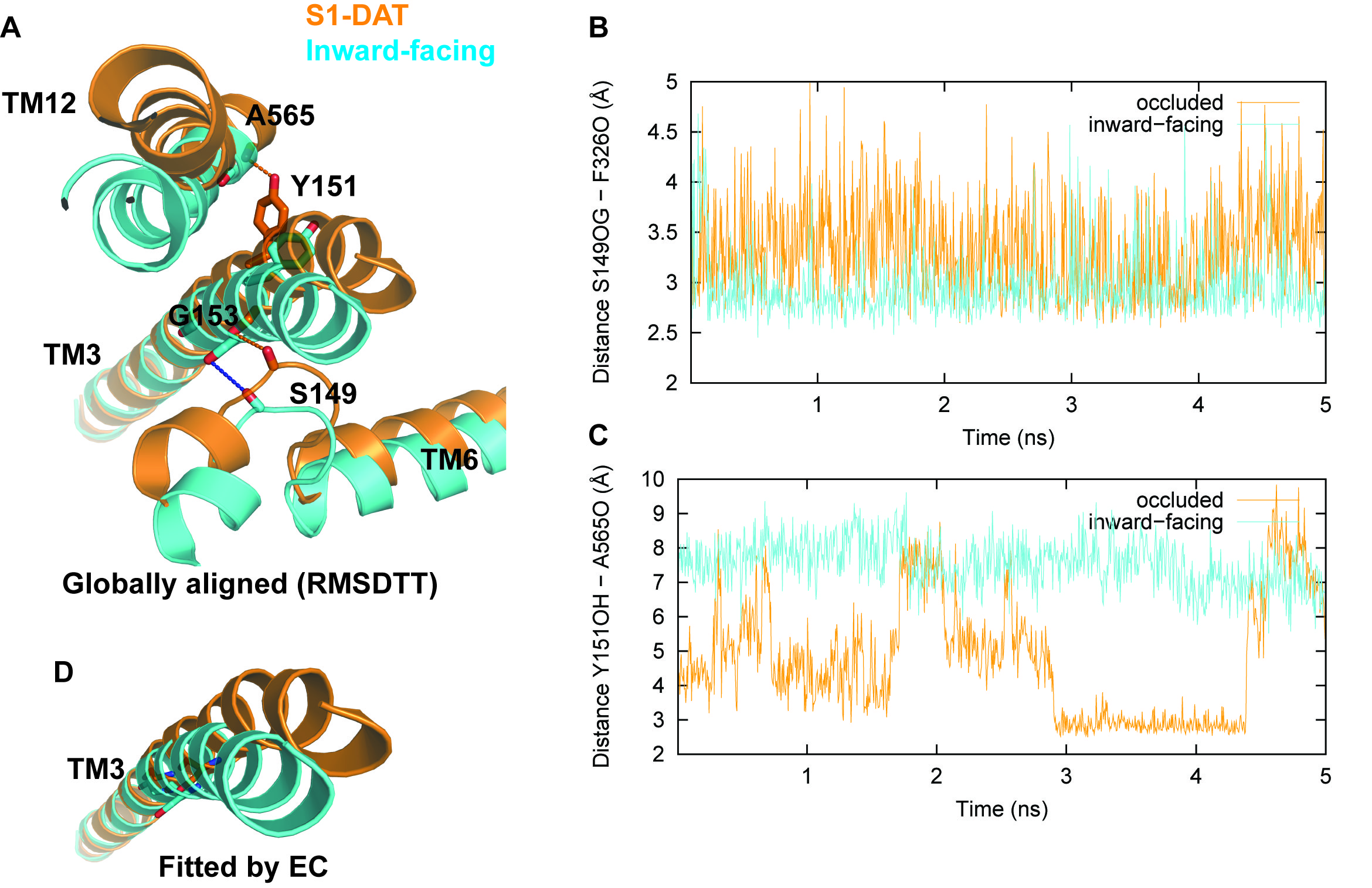


(**A**) Conformational changes in TM3, 6 and 12 from S1-DAT (orange) to the inward-facing conformation (cyan). DAT was aligned with RMSDTT using the entire protein. The intracellular segment of TM3 moved to the place originally occupied by TM6, and TM12 moves toward TM3. The H-bond between S149^3.43^ and F326^6.59^ is maintained (**B**); the one between A565^12.42^ and Y151^3.45^ is broken (**C**).
(**D**) TM3 in the inward-facing conformation is fitted to that in the occluded conformation using the extracellular segment F154^3.48^ – T172^3.66^.

**Figure S7. Conformational changes associated with TM6.**
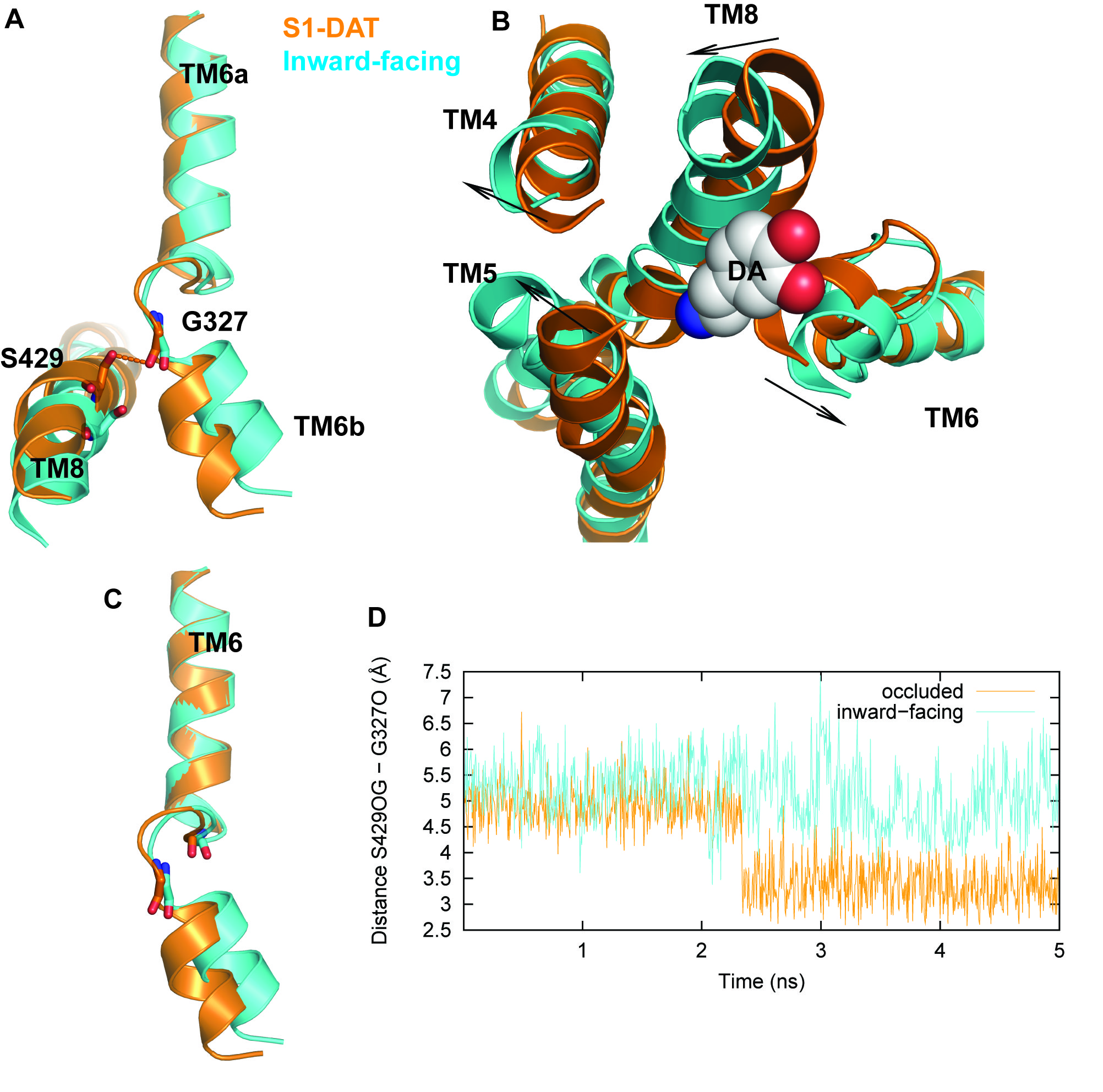


(**A**) TM6b moved away from TM8 in the inward-facing conformation (cyan) compared to S1-DAT (orange). The H-bond between G327^6.60^ and S429^8.67^ is broken (**D**). DAT was aligned with RMSDTT using the whole structure.
(**B**) The intracellular segments of TMs4, 5, and 8 moved outward and that of TM6 moved outward in the opposite direction, creating space for the exiting DA substrate molecule.
(**C**) TM6 in the inward-facing conformation is fitted to that in S1-DAT with the EC segment A308^6.41^ – L322^6.55^. (**D**) The distances between the carboxyl oxygen of S429^8.67^ and the carbonyl oxygen of G327^6.60^ in S1-DAT (orange) and the inward-facing conformation (cyan).

**References**

1. Yamashita A, Singh SK, Kawate T, Yan Jin Y, Gouaux E (2005) Crystal structure of a bacterial homologue of Na^+^/Cl^-^ dependent neurotransmitter transporters. Nature 437: 215-223.

2. Gracia L (2005) RMSDTT: RMSD Trajectory Tool. 2.5 ed. Weill Medical College of Cornell University, Department of Physiology and Biophysics.

3. Henry LK, Adkins EM, Han Q, Blakely RD (2003) Serotonin and cocaine-sensitive inactivation of human serotonin transporters by methanethiosulfonates targeted to transmembrane domain I. J Biol Chem 278: 37052-37063.

4. Zhou Y, Bennett ER, Kanner BI (2004) The aqueous accessibility in the external half of transmembrane domain I of the GABA transporter GAT-1 Is modulated by its ligands. J Biol Chem 279: 13800-13808.

5. Beuming T, Shi L, Javitch JA, Weinstein H (2006) A comprehensive structure-based alignment of prokaryotic and eukaryotic neurotransmitter/Na+ symporters (NSS) aids in the use of the LeuT structure to probe NSS structure and function. Mol Pharmacol 70: 1630-1642.

6. Lin Z, Itokawa M, Uhl GR (2000) Dopamine transporter proline mutations influence dopamine uptake, cocaine analog recognition, and expression. FASEB J 14: 715-728.

7. Itokawa M, Lin Z, Uhl GR (2002) Dopamine efflux via wild-type and mutant dopamine transporters: alanine substitution for proline-572 enhances efflux and reduces dependence on extracellular dopamine, sodium and chloride concentrations. Brain Res Mol Brain Res 108: 71-80.

8. Lin Z, Uhl GR (2005) Proline mutations induce negative-dosage effects on uptake velocity of the dopamine transporter. J Neurochem 94: 276-287.

9. Sen N, Shi L, Beuming T, Weinstein H, Javitch JA (2005) A pincer-like configuration of TM2 in the human dopamine transporter is responsible for indirect effects on cocaine binding. Neuropharmacology 49: 780-790.

10. Sucic S, Bryan-Lluka LJ (2005) Roles of transmembrane domain 2 and the first intracellular loop in human noradrenaline transporter function: pharmacological and SCAM analysis. J Neurochem 94: 1620-1630.

11. Sato Y, Zhang YW, Androutsellis-Theotokis A, Rudnick G (2004) Analysis of transmembrane domain 2 of rat serotonin transporter by cysteine scanning mutagenesis. J Biol Chem 279: 22926-22933.

12. Lopez-Corcuera B, Nunez E, Martinez-Maza R, Geerlings A, Aragon C (2001) Substrate-induced conformational changes of extracellular loop 1 in the glycine transporter GLYT2. J Biol Chem 276: 43463-43470.

13. Zhou Y, Kanner BI (2005) Transporter-associated currents in the gamma-aminobutyric acid transporter GAT-1 are conditionally impaired by mutations of a conserved glycine residue. J Biol Chem 280: 20316-20324.

14. Mao Y, Mathewson L, Gesmonde J, Sato Y, Holy M, et al. (2008) Involvement of serotonin transporter extracellular loop 1 in serotonin binding and transport. Mol Membr Biol 25: 115-127.

15. Singh SK, Piscitelli CL, Yamashita A, Gouaux E (2008) A Competitive Inhibitor Traps LeuT in an Open-to-Out Conformation. Science 322: 1655-1661.

16. Dodd JR, Christie DL (2005) Substituted cysteine accessibility of the third transmembrane domain of the creatine transporter: defining a transport pathway. J Biol Chem 280: 32649-32654.

17. Kleinberger-Doron N, Kanner BI (1994) Identification of tryptophan residues critical for the function and targeting of the gamma-aminobutyric acid transporter (subtype A). J Biol Chem 269: 3063-3067.

18. Lin Z, Zhang PW, Zhu X, Melgari JM, Huff R, et al. (2003) Phosphatidylinositol 3-kinase, protein kinase C, and MEK1/2 kinase regulation of dopamine transporters (DAT) require N-terminal DAT phosphoacceptor sites. J Biol Chem 278: 20162-20170.

19. Golovanevsky V, Kanner BI (1999) The reactivity of the gamma-aminobutyric acid transporter GAT-1 toward sulfhydryl reagents is conformationally sensitive. Identification of a major target residue. J Biol Chem 274: 23020-23026.

20. Itokawa M, Lin Z, Cai NS, Wu C, Kitayama S, et al. (2000) Dopamine transporter transmembrane domain polar mutants: ΔG and ΔΔG values implicate regions important for transporter functions. Mol Pharmacol 57: 1093-1103.

21. Govaerts C, Blanpain C, Deupi X, Ballet S, Ballesteros JA, et al. (2001) The T*X*P motif in the second transmembrane helix of CCR5: A structural determinant of chemokine-induced activation. J Biol Chem: 13217-13225.

22. Zhang YW, Rudnick G (2005) Cysteine-scanning mutagenesis of serotonin transporter intracellular loop 2 suggests an alpha-helical conformation. J Biol Chem 280: 30807-30813.

23. Bryan-Lluka LJ, Bonisch H, Lewis RJ (2003) chi-Conopeptide MrIA partially overlaps desipramine and cocaine binding sites on the human norepinephrine transporter. J Biol Chem 278: 40324-40329.

24. Prasad HC, Zhu CB, McCauley JL, Samuvel DJ, Ramamoorthy S, et al. (2005) Human serotonin transporter variants display altered sensitivity to protein kinase G and p38 mitogen-activated protein kinase. Proc Natl Acad Sci U S A 102: 11545-11550.

25. Mari SA, Soragna A, Castagna M, Santacroce M, Perego C, et al. (2006) Role of the conserved glutamine 291 in the rat gamma-aminobutyric acid transporter rGAT-1. Cell Mol Life Sci 63: 100-111.

26. Hastrup H, Karlin A, Javitch JA (2001) Symmetrical dimer of the human dopamine transporter revealed by cross-linking Cys-306 at the extracellular end of the sixth transmembrane segment. Proc Natl Acad Sci U S A 98: 10055-10060.

27. Zhou Y, Zomot E, Kanner BI (2006) Identification of a lithium interaction site in the gamma-aminobutyric acid (GABA) transporter GAT-1. J Biol Chem 281: 22092-22099.

28. Keller PC, 2nd, Stephan M, Glomska H, Rudnick G (2004) Cysteine-scanning mutagenesis of the fifth external loop of serotonin transporter. Biochemistry 43: 8510-8516.

29. Plenge P, Wiborg O (2005) High- and low-affinity binding of S-citalopram to the human serotonin transporter mutated at 20 putatively important amino acid positions. Neurosci Lett 383: 203-208.
